# Conventional vs. Microwave- or Mechanically-Assisted Synthesis of Dihomooxacalix[4]arene Phthalimides: NMR, X-ray and Photophysical Analysis ^§^

**DOI:** 10.3390/molecules26061503

**Published:** 2021-03-10

**Authors:** Alexandre S. Miranda, Paula M. Marcos, José R. Ascenso, M. Paula Robalo, Vasco D. B. Bonifácio, Mário N. Berberan-Santos, Neal Hickey, Silvano Geremia

**Affiliations:** 1 Centro de Química Estrutural, Faculdade de Ciências da Universidade de Lisboa, Edifício C8, 1749-016 Lisboa, Portugal; miranda.m.alexandre@gmail.com; 2 IBB-Institute for Bioengineering and Biosciences, Instituto Superior Técnico, Universidade de Lisboa, 1049-001 Lisboa, Portugal; vasco.bonifacio@tecnico.ulisboa.pt (V.D.B.B.); berberan@tecnico.ulisboa.pt (M.N.B.-S.); 3 Faculdade de Farmácia, Universidade de Lisboa, Av. Prof. Gama Pinto, 1649-003 Lisboa, Portugal; 4 Centro de Química Estrutural, Instituto Superior Técnico, Complexo I, Av. Rovisco Pais, 1049-001 Lisboa, Portugal; jose.ascenso@ist.utl.pt (J.R.A.); mprobalo@deq.isel.ipl.pt (M.P.R.); 5 Área Departamental de Engenharia Química, Instituto Superior de Engenharia de Lisboa, Instituto Politécnico de Lisboa, Rua Conselheiro Emídio Navarro, 1, 1959-007 Lisboa, Portugal; 6 Centre of Excellence in Biocrystallography, Department of Chemical and Pharmaceutical Sciences, University of Trieste, via L. Giorgieri 1, 34127 Trieste, Italy; nhickey@units.it (N.H.); sgeremia@units.it (S.G.)

**Keywords:** dihomooxacalix[4]arenes, phthalimide derivatives, conventional synthesis, microwave irradiation, ball milling, NMR spectroscopy, X-ray diffraction, electronic absorption and fluorescence studies

## Abstract

Direct *O*-alkylation of *p-tert*-butyldihomooxacalix[4]arene (**1**) with *N*-(bromopropyl)- or *N*-(bromoethyl)phthalimides and K_2_CO_3_ in acetonitrile was conducted under conventional heating (reflux) and using microwave irradiation and ball milling methodologies. The reactions afforded mono- and mainly distal di-substituted derivatives in the cone conformation, in a total of eight compounds. They were isolated by column chromatography, and their conformations and the substitution patterns were established by NMR spectroscopy (^1^H, ^13^C, COSY and NOESY experiments). The X-ray structures of four dihomooxacalix[4]arene phthalimide derivatives (**2a**, **3a**, **3b** and **5a**) are reported, as well as their photophysical properties. The microwave (MW)-assisted alkylations drastically reduced the reaction times (from days to less than 45 min) and produced higher yields of both 1,3-di-substituted phthalimides (**3a** and **6a)** with higher selectivity. Ball milling did not reveal to be a good method for this kind of reaction.

## 1. Introduction

Calixarene-based molecules are amongst the most investigated frameworks in host-guest and supramolecular chemistry [1,2]. Their relatively easy functionalization at either the upper or lower rim, a pre-organized cavity available in different sizes and conformations, and ion-binding sites are the key for their great diversity of applications. The recognized importance of anions in biological, chemical and environmental processes continues to attract chemists for the design and use of new synthetic anion receptors [3,4]. Calixarenes possessing (thio)urea moieties have been developed as receptors, with anion interactions established by hydrogen bonding only.

Since the first articles on the use of domestic microwave ovens for organic synthesis in 1986 [5,6], this technology has become a very important tool in a wide range of chemical reactions [7,8,9,10]. The microwave (MW) technique provides uniform heating of the reagents throughout the reaction vessel, allowing a rapid and homogeneous heat transfer. MW heating drastically reduces reaction times compared to conventional heating (from hours or even days to some minutes), saving a huge amount of energy and time. Higher product yields, higher reaction selectivity and low waste are other advantages of this technique, as well as the use of less or even no solvent and catalyst. Ball milling (BM), another environmentally friendly methodology applied to organic synthesis under solvent-free conditions, has also been developed mainly in the last decade [11,12,13]. Both techniques have been employed in the synthesis of calixarenes and related macrocyclic hosts [14], although ball milling is much less extensive.

The syntheses of *p*-alkylcalix[*n*]arenes (R = *t*-Bu, *n* = 4 and 8; R = Me, *n* = 6) were the first calixarene reactions carried out under MW irradiation [15,16]. Other examples reported are the preparation of water-soluble azocalix[4]arenes [17], the copper(I)-catalysed syntheses of calix[4]arene glycoconjugates [18] and calix[4]arene tetraazide derivatives [19], as well as calix[4]arenes containing chiral unsymmetrical urea moieties at the lower rim [20]. The alkylation of *p-tert*-butylcalix[4 and 6]arenes in the presence of K_2_CO_3_ and MeCN or DMF are among the most investigated MW-assisted reactions [21,22,23]. Calix[6]arenes functionalized at the lower and upper rims were also successfully obtained [24]. All these reactions were shown to be faster and more efficient compared to conventional methods. The formation of *p*-benzylcalix[5 and 7]arenes from *p*-benzylcalix[6 or 8]arenes in the presence of KOH, formaldehyde and molecular sieves [25], and the synthesis of a *p*-nitrocalix[6]arene [26] are the only examples of ball milling methods employed in calixarene synthesis found in the literature.

In the course of our research on anion binding by ureido-dihomooxacalix[4]arenes [27,28,29,30,31], we were interested in studying the introduction of urea binding sites into the 1,3-positions of the calixarene lower rim, through shorter spacers (propyl or ethyl) compared to the ones obtained in our previous dihomooxa receptors. The two remaining phenolic OH groups will participate in the formation of hydrogen bonds, contributing to keep the macrocycle in the cone conformation. It is known that *O*-alkylation reactions of calixarenes by conventional methods can take a long time and produce low yield products. Thus, MW irradiation and ball milling were employed in this work to obtain the target 1,3-disubstituted phthalimides, which have been prepared as precursors to urea-terminated anion-binding macrocycles. Other differently substituted derivatives were also obtained. Their solution and solid state conformational analysis and the determination of some photophysical properties are also reported.

## 2. Results and Discussion

### 2.1. Conventional vs. MW- and Mechanically-Assisted Synthesis

**Scheme 1:**
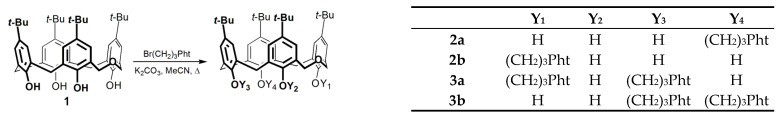
Synthesis of propylphthalimide derivatives **2** and **3**.

With the aim of introducing urea groups at the 1,3-positions of the *p-tert*-butyldihomooxacalix[4]arene (**1**) via a propyl spacer, the parent compound **1** was treated with 2 equiv of *N*-(3-bromopropyl)phthalimide and 2 equiv of K_2_CO_3_ in refluxing acetonitrile for 4 days (Scheme 1). This reaction afforded, according to the proton NMR spectrum, a mixture composed of distal 1,3-diphthalimide **3a** as the major product (65%), along with minor amounts of mono **2a** (13%) and tetra-phthalimide **4** (12%), and a further 3% of proximal 3,4-diphthalimide **3b** and 6% of unreacted **1** (Table 1, entry 1). A trace amount of mono **2b** was also observed. When the reaction was conducted for 5 days, the percentage of formation of **3a** increased (76%), as well as that of **3b** (15%), while minor amounts of tetra **4**, mono **2a** and no starting material were obtained (Table 1, entry 2).

In view of the long reaction time, the alkylation of parent **1** was investigated under MW irradiation and ball milling methodologies. The reaction of **1** with 2 equiv of the previous alkylating agent and base in acetonitrile after 20 min of irradiation (160 °C) afforded 86% of 1,3-diphthalimide **3a**, accompanied by 10% of mono **2a** and minor amounts (<2%) of proximal di-phthalimide **3b** and tetra **4** (Table 1, entry 3). The increase in the irradiation time to 30 min (Table 1, entry 4) produced an increase of 1,3-diphthalimide **3a** (93%) and a decrease of mono **2a** (3%). The use of solvent-free ball milling during 2 h with a rotation speed of 500 rpm, 200 stainless steel balls and keeping the same reagent ratio as before gave a mixture composed of 70% of unreacted **1** and 30% of mono **2a** (Table 1, entry 5). Replacing stainless steel balls with zirconium oxide balls and increasing the rotation speed to 650 rpm led to a similar result. After several attempts to improve the reaction yield, we found that using 4 equiv of alkylating agent and base, and prolonging the reaction time to 12 h, raised the yield of mono **2a** to 46%, although 27% of the starting material was still present (Table 1, entry 6). The formation of the previous distal and proximal diphthalimides **3a** and **3b**, as well as of the tetra derivative **4**, was also observed, with distal **3a** being obtained as the main compound (19%).

The separation of the reaction mixture into the pure compounds was achieved by column chromatography and by recrystallization. The tetra-phthalimide derivative **4** was not purified, as it had already been obtained by us before [29].

**Scheme 2:**
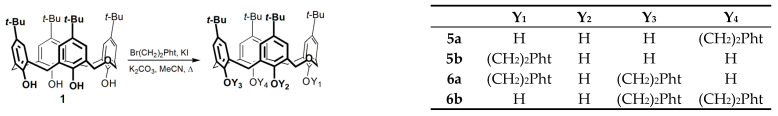
Synthesis of ethylphthalimide derivatives **5** and **6**.

Concerning the synthesis of the 1,3-diethylphthalimide **6a** (Scheme 2), a urea precursor with an even shorter spacer (only two carbon atoms), several difficulties were found in its preparation. The alkylation of parent **1** with *N*-(2-bromoethyl)phthalimide and K_2_CO_3_ was tried under different conditions. Firstly, the reaction was carried out using 2 equiv of the reactant and base for 3 days in acetonitrile under reflux. According to the proton NMR integration, a mixture composed mainly of unreacted **1** (61%) and mono-phthalimide **5a** (21%), along with minor amounts of mono **5b** (8%) and 1,3-diphthalimide **6a** (11%), was obtained (Table 2, entry 1). When the reaction was conducted for the same time, but in the presence of KI (to promote the halogen exchange of the electrophile in situ) and a 25% excess of alkylating agent, the percentage of unreacted **1** decreased (30%) and those of mono **5a** (45%) and 1,3-di **6a** increased (16%), although the latter only slightly (Table 2, entry 2). To improve the reactivity of the nucleophile, **1** was also previously heated with the base in MeCN for about 30 min. Four equiv of reactant and base were necessary to raise the percentage of **6a** to 30% and to decrease that of **1** to 19% (Table 2, entry 3). A final attempt under extreme conditions (6 equiv of Br(CH_2_)_2_Pht and base for 7 days in refluxing MeCN) gave identical results (Table 2, entry 4). Substitution and elimination reactions are almost always in competition with each other. The peak assignments in the proton NMR spectrum of the reaction mixture compatible with bromo- and iodo-ethylphthalimides, as well as *N*-ethenylphthalimide in an approximately 40:60 ratio, indicate that elimination should predominate over substitution, explaining the low yields obtained even under extreme conditions [32].

The alkylation reaction with *N*-(2-bromoethyl)phthalimide was also studied by MW irradiation, under a range of conditions. The irradiation of parent **1** with 2 equiv of base in acetonitrile for 6 min at 110 °C, followed by the addition of 4 equiv of reagent and 2 equiv of KI and a further 12 min irradiation at 150 °C, afforded a mixture of mono **5a** as the main product, along with 1,3-di **6a** and unreacted **1** (Table 2, entry 5). An increase in the irradiation time at 150 °C (36 min) led to the disappearance of **1** and to an increase of **6a** (Table 2, entry 6). The effect of KI on the reaction yield was investigated in two MW-assisted alkylations carried out with and without KI (Table 2, entries 7 and 8, respectively). Given that just a slight improvement (5%) of **6a** yield was obtained in the presence of KI, the next assay was performed without KI, but with 4 equiv of base, an irradiation time of 18 min (110 °C), followed by a further 36 min (150 °C), resulting **6a** as the unique product (Table 2, entry 9). The reduction of the time at 110 °C to 6 min provided 1,3-diphthalimide **6a** as the unique product as well (Table 2, entry 10). As expected, the MW-assisted reactions showed several advantages compared to the conventional synthesis, mainly in the case of the alkylation with *N*-(bromoethyl)phthalimide. Beyond the removal of KI reactant and the reduction in solvent quantity, the MW irradiation provided drastically shorter reaction times. The alkylation of parent **1** was complete after 42 min under MW conditions, while under reflux for 7 days the conversion was still incomplete. Higher reaction selectivity was also observed, with distal 1,3-disubstituted phthalimide **6a** being the only derivative obtained. Ball milling was likewise performed, but gave the poorest results (Table 2, entry 11).

It was not possible to isolate the mono-phthalimide **5b** as a pure compound, despite the several columns and preparative chromatographies done. Recrystallization attempts in different solvents have also failed. Similarly, distal 1,3-diphthalimide **6a** and proximal 3,4-diphthalimide **6b** could not be separated from each other, as they have the same R_f_ value in all the solvents tested. A similar situation was reported before for distal and proximal di-substituted calix[4]arene derivatives [19].

Besides the NMR assignment of all the previous derivatives, the proton spectra of both reaction mixtures still present a set of small signals, which could not be identified. Since a total of nine possible *O*-alkylating compounds can be obtained [33], the formation of other differently substituted phthalimide derivatives cannot be excluded.

### 2.2. NMR Conformational Analysis

The conformation and the substitution pattern of the phthalimide derivatives were established by proton, carbon-13, COSY and NOESY NMR spectroscopy in chloroform at room temperature.

#### 2.2.1. Mono-Propylphthalimide **2a**

**Figure 1:**
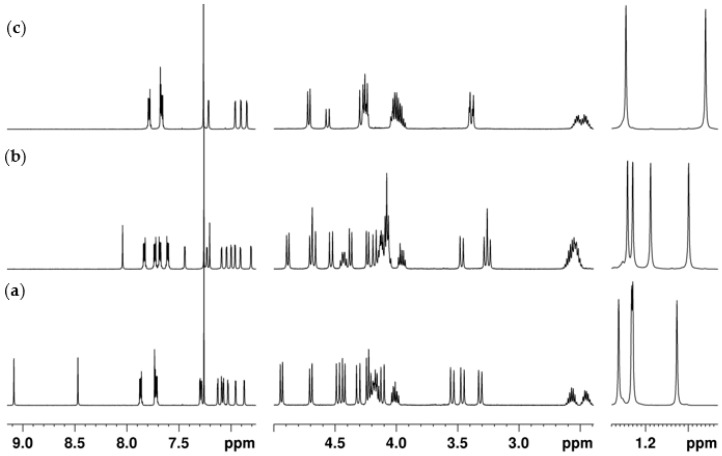
^1^H NMR spectra (500 MHz, CDCl_3_, 22 °C) of compounds: (**a**) **2a**, (**b**) **3a** and (**c**) **3b**.

The absence of symmetry in compound **2a** is reflected by its proton and carbon-13 NMR spectra. The ^1^H NMR spectrum (Figure 1a) displays four singlets for the *tert*-butyl groups, five AB quadruplets for the CH_2_ bridge protons, four pairs of doublets for the aromatic protons and three singlets for the OH groups of the calixarene skeleton, besides several multiplets for the -OCH_2_CH_2_CH_2_N- methylene protons and phthalimide group (two signals). The proton assignments were confirmed by cross-peak correlations in a COSY spectrum. The ^13^C NMR spectrum shows 28 downfield resonances arising from the aromatic carbon atoms and phthalimide group, three downfield resonances arising from the methylene carbon atoms of -O***C***H_2_CH_2_CH_2_N- and CH_2_OCH_2_ groups, and 12 of the expected 13 upfield resonances arising from the quaternary (3 lines in a 1:1:2 ratio) and methyl carbon atoms (4 lines) of the *tert*-butyl groups, and the methylene carbon atoms of the ArCH_2_Ar (3 lines) and -OCH_2_***C***H_2_***C***H_2_N- (2 lines) groups. All resonances were assigned by DEPT experiments. The three ArCH_2_Ar resonances appear at 30.1, 31.3, and 32.8 ppm, indicating a cone conformation for **2a** [34].

**Figure 2:**
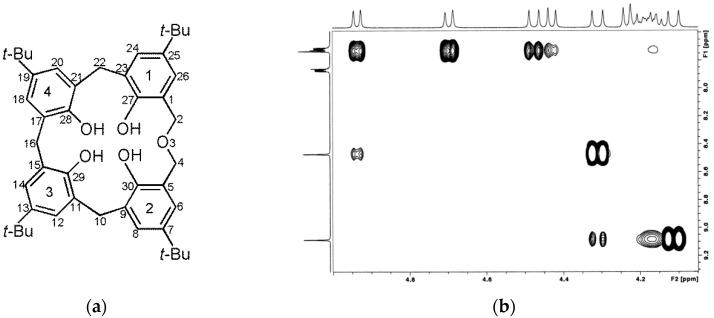
(**a**) Chemical structure of **1** with carbon atoms numbering; (**b**) Section of the NOESY spectrum of **2a** (500 MHz, CDCl_3_, 22 °C).

The position of the phthalimide group was confirmed by proton-proton correlations observed in the NOESY spectrum. The preferential formation of **2a** over **2b** is expected, as described by us before [33]. The simultaneous observation of two NOE effects between the OH_1_ proton at 9.08 ppm (position 29) and the two axial methylene protons at positions 10 and 16, as well as those observed between the OH_2_ group at 8.47 ppm (position 30) and the axial CH_2_ protons at positions 4 and 10, and between the OH_3_ group at 7.73 ppm (position 27) and the axial CH_2_ protons at positions 2, 22 and 4, are conclusive for the position of the substituent group (Figure 2). In addition to those effects, two other relevant NOEs were observed between OH_1_ and -OCH_2_CH_2_CH_2_N- groups and OH_1_ and OH_2_ groups.

Concerning mono-propylphthalimide **2b**, it could be identified as a trace compound in a set of fractions obtained by column chromatography (Figure S1, Table S1).

##### 2.2.2. 1,3-Dipropylphthalimide **3a**

The ^1^H NMR spectrum of **3a** (Figure 1b) is also asymmetric and shows identical number and type of signals as **2a** for the majority of the protons of the calixarene skeleton. Two singlets for the OH groups and four multiplets for the phthalimide aromatic protons can, in addition, be observed. The ^13^C NMR spectrum displays 32 downfield, four midfield and 13 of the expected 15 upfield resonances. The presence of the three ArCH_2_Ar resonances in the range 29.8–32.9 ppm indicates a cone conformation for **3a**.

The 1,3-substitution pattern was also confirmed by a NOESY experiment. The preferential formation of distal disubstituted compounds is expected, as previously reported for the formation of other dihomooxacalix[4]arenes [28,33]. Two simultaneous NOE effects between OH_1_ proton at 8.04 ppm (position 29) and the two axial methylene protons at positions 10 and 16 were observed. Moreover, both OH groups (at 8.04 and 7.20 ppm) give NOE effects on both -OCH_2_CH_2_CH_2_N- groups (positions 28 and 30), and OH_2_ (position 27) also gives effects on the axial CH_2_ protons at positions 2 and 22.

#### 2.2.3. 3,4-Dipropylphthalimide **3b**

Derivative **3b** exhibits symmetric proton and carbon-13 NMR spectra, matching with a proximal disubstituted compound in the cone conformation. The ^1^H NMR spectrum (Figure 1c) shows two singlets for the *tert*-butyl groups, three AB quadruplets in a 2:2:1 ratio for the CH_2_ bridge protons, two pairs of doublets for the aromatic protons, one singlet for the OH groups, four multiplets for the -OCH_2_CH_2_CH_2_N- and two multiplets for the phthalimide groups. The ^13^C NMR spectrum displays 16 downfield, two midfield and 8 upfield resonances. The ArCH_2_Ar resonances appear at 30.8 (one carbon atom) and 31.6 (two carbon atoms), indicating a cone conformation for **3b**.

The positions of the phthalimide groups were confirmed by two simultaneous NOE effects observed between the -OCH_2_CH_2_CH_2_N- protons (position 28) and the two axial CH_2_ protons at positions 16 and 22, as well as between the OH proton (position 27) and the axial CH_2_ proton at position 2.

#### 2.2.4. Mono-Ethylphthalimide **5a**

**Figure 3:**
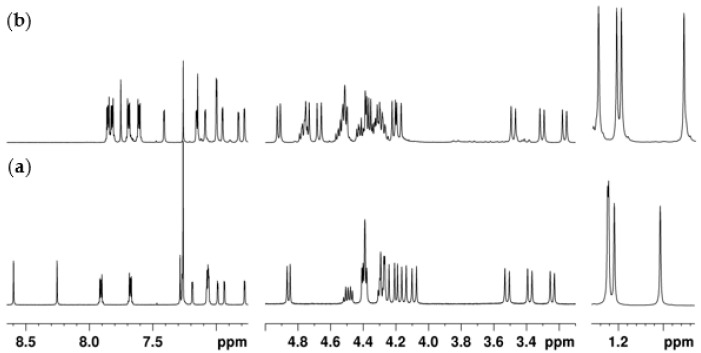
^1^H NMR spectra (500 MHz, CDCl_3_, 22 °C) of compounds: (**a**) **5a** and (**b**) **6a**.

Similarly to mono-propylphthalimide **2a**, derivative **5a** also presents asymmetric proton (Figure 3a) and carbon-13 spectra, being the lower number of multiplets for the CH_2_ protons of the pendant group the only observed difference. The same 12 upfield carbon resonances are observed (as in mono **2a**), as there is no overlapping of the quaternary carbon atoms (4 lines) of the *tert*-butyl groups.

Concerning the phthalimide group position, the NOESY spectrum of **5a** shows identical effects to those exhibited for **2a**. NOEs between each of the three OH groups (position 29, 30 and 27) and the two adjacent axial CH_2_ protons (positions 10/16, 4/10 and 2/22, respectively) are observed. Furthermore, the -OCH_2_CH_2_N- group gives NOE effects on both OH_1_ and OH_3_ protons (positions 29 and 27), and each OH group gives NOE on its OH neighbor (OH_1_ ↔ OH_2_ ↔ OH_3_).

A pure mixture of mono-phthalimides **5b** and **5a** was obtained by preparative chromatography (Figure S1, Table S1). However, further separation of mono **5b** from the mixture was not possible to achieve.

#### 2.2.5. 1,3- and 3,4-Diethylphthalimides **6a** and **6b**

As mentioned before, 1,3-diphthalimide **6a** and 3,4-diphthalimide **6b** could not be separated from each other, as they have the same R_f_ value. In the case of the MW-assisted reaction, a 100% conversion of parent **1** into **6a** was obtained. However, after several purification attempts it was not possible to obtain pure **6a**, as revealed by the ^1^H spectrum (Figure 3b).

Both ^1^H and ^13^C NMR spectra of **6a** show spectral patterns compatible with an asymmetric molecule and very similar to those obtained for **3a**. The presence of the three ArCH_2_Ar carbon-13 resonances at 29.8, 30.0 and 32.7 ppm indicates a cone conformation for **6a**. The 1,3-substitution was also corroborated by proton-proton correlations observed in the NOESY spectrum. Concerning derivative **6b**, it was possible to clearly identify in the ^1^H spectrum two singlets for the *tert*-butyl groups, two pairs of doublets for the aromatic protons and one singlet for the OH groups (Figure S1, Table S1). The overlapping of signals (with those of **6a**) prevents the assignment of the methylenic region.

### 2.3. X-ray Structural Analysis

**Figure 4:**
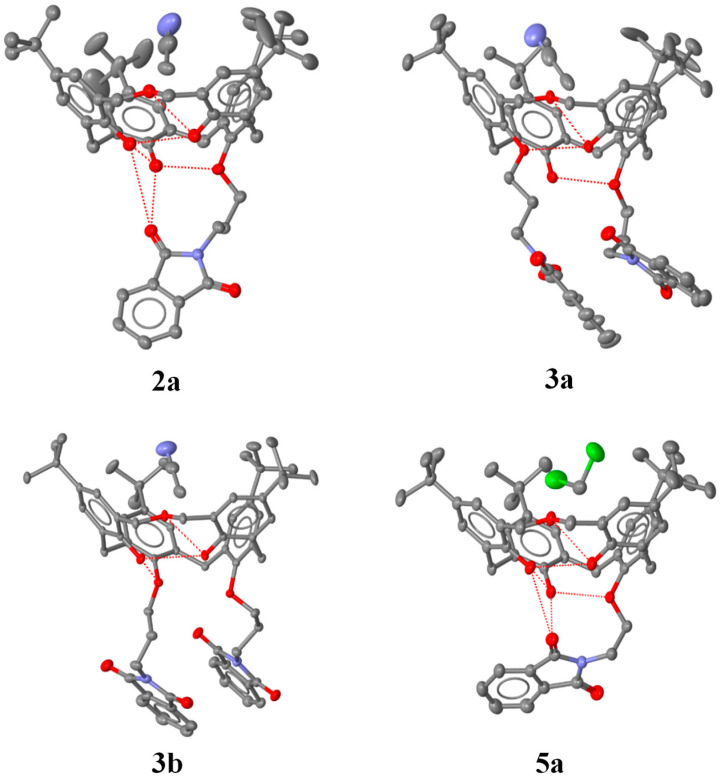
Thermal ellipsoid representations of **2a**, **3a**, **3b** and **5a**. Only one of the crystallographically independent molecules is shown for **2a** (4 molecules in the asymmetric unit) and for **3a** (3 molecules in the asymmetric unit). The atomic species are represented in CPK colors. Ellipsoids are drawn at 50% probability, except for **2a**, in which the ellipsoids are represented at 30% probability. Hydrogen atoms, disordered fragments and solvent molecules located outside the calixarene cavities are omitted for clarity.

Small single crystals of the mono-substituted **2a** and **5a** and the di-substituted **3a** and **3b** were analyzed by X-ray diffraction at 100 K using synchrotron radiation. With the exclusion of the symmetric **3b**, all the other molecules are inherently chiral due to the presence of the phthalimide substituents on the lower rim, which are not located symmetrically with respect to the oxa bridge inserted in the calixarene cone (Figure 4). As all their space groups are centrosymmetric (C2/c for **2a** and **3a** and P-1 for **5a**), racemic mixtures of the two inherently chiral enantiomers are present in these crystals.

**Figure 5:**
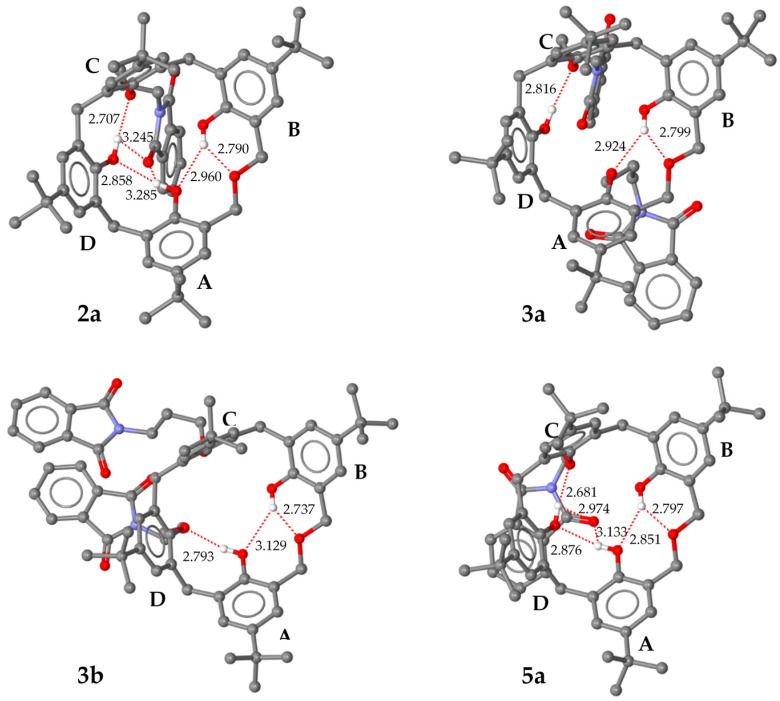
Comparison of the intramolecular hydrogen bonds in **2a**, **3a**, **3b** and **5a**. Only one of the crystallographically independent molecules is shown for **2a** (4 molecules in the asymmetric unit) and for **3a** (3 molecules in the asymmetric unit). The atomic species are represented in CPK colors. Ellipsoids are drawn at 50% probability, except for **5a**, in which the ellipsoids are represented at 30% probability. Hydrogen atoms, atom disorder and solvent molecules are omitted for clarity.

A different number of crystallographically independent molecules was observed for the analyzed structures: four for **2a**, three for **3a**, and one for **3b** and **5a**. In order to compare the different structures in a coherent manner, the asymmetric units with all the molecules having the same inherent chirality were selected. The same labeling scheme was applied to these molecules, namely, the phenyl ring labels A-D are ordered anti-clockwise, as viewed from above the calix cone, with A and B across the oxa bridge. In this scheme, ring C always has a phthalimide substituent and ring B always has a hydroxyl group, while rings A and D have a hydroxyl group (**2a** and **5a**), except for the di-substituted derivatives, in which the second phthalimide substituent is present on ring A (**3a**) or ring D (**3b**) (Figure 5).

**Figure 6:**
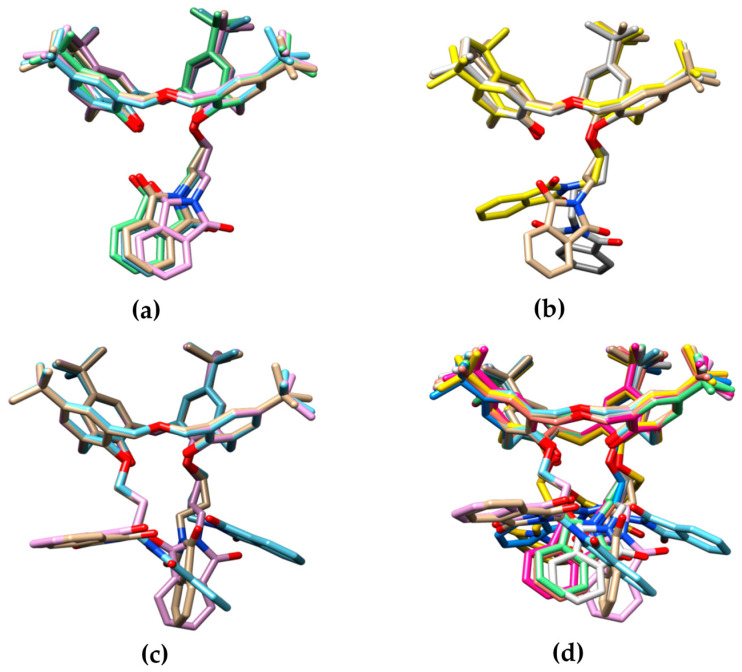
Comparison of the conformations of the phthalimide arms. All molecules exhibit very similar cone conformations: (**a**) Overlay of the 4 independent molecules of **2a**; (**b**) Overlay of **5a** (ethyl linker) one of the 4 independent molecules of **2a** (propyl linker) and the analogous derivative with butyl linker [35]; (**c**) Overlay of the 3 independent molecules of **3a**; (**d**) Overlay of the 9 independent molecules of **2a**, **3a**, **3b** and **5a**. The atomic species are represented in CPK colors. Hydrogen atoms, atom disorder and solvent molecules are omitted for clarity.

The X-ray structures show that all the dihomooxacalix[4]arenes adopt comparable cone conformations (Table 3 and Figure 6). All the canting angles, defined as the dihedral angles between the mean planes of the phenyl rings and the mean plane of the five methylene bridging carbon atoms, lean outwards from the center of the cone with similar values for corresponding phenyl rings, irrespective of the substituent pattern on the lower rim and the consequent differences in the intramolecular H-bond network (Figure 5). In particular, the two phenyl rings adjacent to the oxa bridge show large canting angles (mean values with standard deviation in parenthesis: A = 138(5)° and B = 148(3)°), while the other two are smaller (C = 102(4)° and D = 123(6)°) (see Table 3). This observation indicates that the conformation of the cone in these compounds is not determined by the different substitution pattern and different intramolecular H-bond networks. However, it should be noted that with respect to the cone conformation, all these structures have in common a bifurcated H-bond between the OH-donor group on ring B and acceptor oxygens atoms of the oxa bridge and the hydroxyl/alkoxy group on ring A (Figure 5, Table S2). This means that in all crystallographically independent molecules, the oxa bridge conformation has the oxygen atom oriented towards the center of the cone. The di-substituted derivatives show an additional intramolecular H-bond involving the second OH donor hydroxyl groups on ring A or D with O-acceptor atoms on ring D or C for **3a** or **3b**, respectively (Figure 5, Table S2). On the other hand, both of these H-bonds (A-D and D-C) are observed in the mono-substituted **2a** and **5a**, which have hydroxyl functions on the A and D rings. However, these are both bifurcated H-bonds, involving an additional O acceptor atom of a phthalimide carbonyl group. These bifurcated H-bonds are quite asymmetric, with the second, phthalimide group interaction characterized by long O•••O distances (Figure 5). Although these interactions are weak, they are important to determine the orientation of the phthalimide arms on the lower rims. For example, it is of interest to note that in **2a**, the four crystallographically independent molecules all have the same orientation of the phthalimide arms (Figure 6a). The intramolecular H-bonds involving the phthalimide groups are weaker in **2a**, in which the linker is longer, with respect to **5a** (propyl vs. ethyl). Interestingly, when the linker is even longer, as in the case of a butyl derivative, this potential intramolecular H-bond interaction with the phthalimide group is completely lost (Figure 6b) [35].

**Figure 7:**
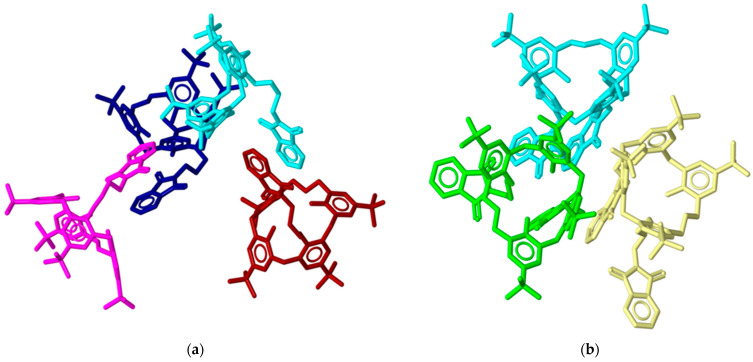
Illustration of intermolecular π-stacking interactions observed in the crystal packing of **2a** (**a**) and **3a** (**b**). In **2a**, the molecules are present as dimeric species through π-stacking of the phthalimide groups of the 4 independent molecules (violet-blue and cyan-brown). In **3a**, in addition to an intramolecular π-stacking interaction observed in one of the independent molecules (cyan), there is an intermolecular π-stacking interaction between the other two (green-yellow). For clarity, independent molecules are represented in arbitrarily chosen different colors, while hydrogen atoms, atom disorder and solvent molecules are omitted.

The effect on the phthalimide arm orientation of the different linkers is also evidenced by the different α angles which the planes of the phthalimide groups make with the mean planes of the five calixarene methylene groups (Figure 6b and Table S3). For **2a** it is almost orthogonal (mean value 77°), while for **5a** it is more parallel (22°). With regard to the di-substituted derivatives **3a** and **3b**, the presence of the second phthalimide arm in place of a hydroxyl group reduces the number of H-bond donors, which reduces the overall strength of potential H-bond interactions with the phthalimide arms. The loss of these interactions increases the conformational freedom of the substituents on the lower rim (Figure 6c,d). In fact, for **3a**, the three crystallographically independent molecules exhibit three different conformations (Figure 6c, Table S3). The presence of a second phthalimide group allows the possibility of intramolecular π-stacking interactions between these aromatic groups, as observed for **3b** and for one crystallographically independent molecule of **3a** (Figure 4 and Figure 6c). On the other hand, intermolecular π-stacking between two phthalimide groups is also observed for the other two molecules of **3a** and for the mono-substituted **2a** derivative (Figure 7). In **2a**, for example, the four molecules can be described as two dimers formed by the intermolecular π-stacking of phthalimide groups.

The importance of the intramolecular H-bonds network on the open conformation of the calixarene cavity is evidenced by the comparison with the dihomooxa calixarene structures reported with four alkoxy groups on the low rim [28,30,31]. In all structures with four substituents on the lower rim, one phenyl group leans towards the center of the cavity, closing the aperture with the *tert*-butyl groups. In fact, no solvent guest molecules were observed in these crystal structures. On the contrary, in the present structure the presence of hydroxyl groups, responsible for the above described H-bond network, pre-organizes the cavity to host guest molecules. In fact, all the reported molecules contain a solvent guest molecule in the calixarene cavity (Figure 4).

### 2.4. Photophysical Properties

**Figure 8:**
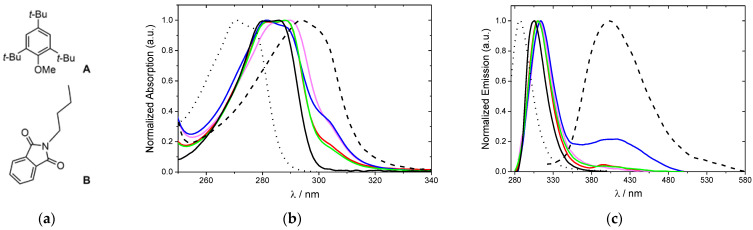
(**a**) Chemical structures of the model compounds **A** and **B**. Normalized absorption (**b**) and emission (**c**) spectra of **1** (black line), **A** (dotted line), **B** (dashed line), **2a** (red line), **3a** (pink line), **3b** (blue line) and **5a** (green line) in CH_2_Cl_2_. [dihomooxa] = 5 × 10^−5^ M; [**A**] = 8 × 10^−4^ M; [**B**] = 2 × 10^−4^ M. $\lambda_{\mathrm{ex}}$ = 270 nm.

Parent calixarenes bear a mesitol-like intrinsic fluorophore. This opens the possibility of using its fluorescence for structural and dynamic information, and for sensing applications [30,31,36]. In the present case, compounds **2a**, **3a**, **3b** and **5a** contain not only mesitol-like and alkylated mesitol-like fluorophores, but also a second chromophore (phthalimide) that can engage in intramolecular electronic energy transfer, acting as acceptor towards the ring fluorophores, as will be shown. To evaluate the changes caused by the introduction of the phthalimide substituent, parent **1** and model compounds 1,3,5-tri-*tert*-butyl-2-methoxybenzene (**A**) and *N*-butylphthalimide (**B**) were also studied. The absorption and emission spectra of **1**, of model compounds and of dihomooxa phthalimide derivatives in dichloromethane are shown in Figure 8. All of them present a well-defined absorption in the ultraviolet region, with phthalimide derivatives slightly red shifted compared to parent **1** and displaying a shoulder at the long wavelength side (Figure 8b). The absorption of **1** is in turn red-shifted with respect to that of **A**. These results indicate that the macrocycle dominates the absorption in the near UVC region (270–290 nm), while the phthalimide groups contribute to the absorption in the UVB region (290–320 nm). Dihomooxa compounds also display a well-defined emission in the UV region (Figure 8c) that extends into the visible owing to the (minor) phthalimide moiety contribution. Indeed, **B** exhibits a broad spectrum peaking at about 400 nm and extending from the UVA to the yellow region, in agreement with data reported for other *N*-phthalimides [37]. The weak 400 nm band is more evident for proximal di-phthalimide **3b**, probably owing to the proximity of the substituent groups at the lower rim. The relevant photophysical properties of all compounds studied are collected in Table 4.

**Figure 9:**
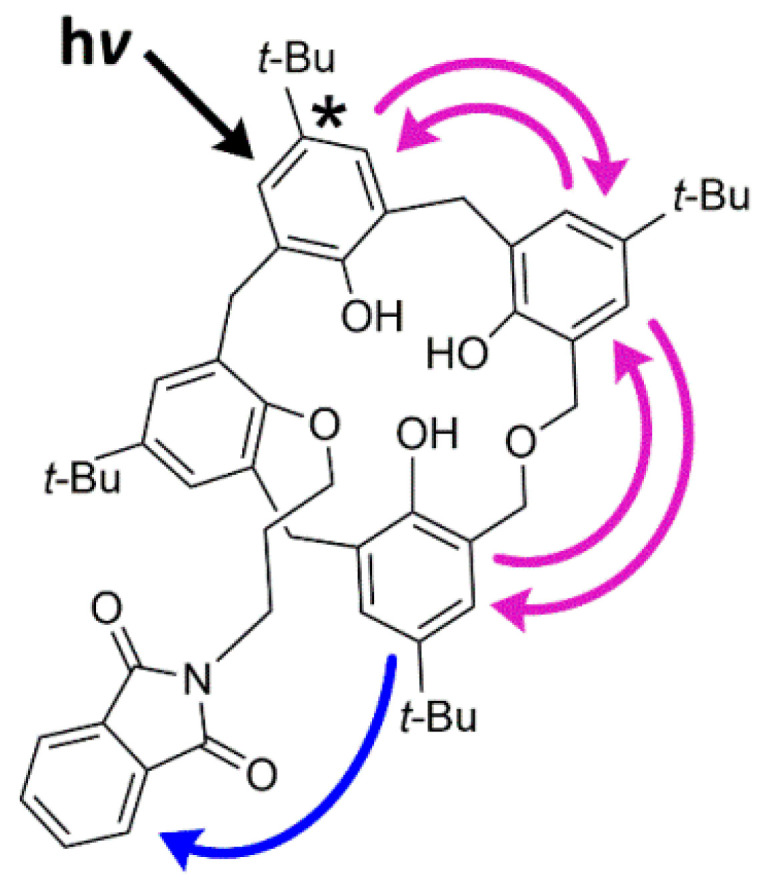
Schematic representation for compound **2a** of homotransfer (reversible FRET) within the calixarene followed by heterotransfer (irreversible FRET) from the calixarene to the phthalimide group (only one of many possible excitation outcomes is shown).

The significant spectral overlap between the absorption and the fluorescence of the calixarene chromophores, on the one hand, and between the absorption of the phthalimides and the fluorescence of the calixarene chromophores, on the other hand (Figure S2), along with the close distance between chromophores, makes possible the existence of resonance energy transfer, both of the homotransfer (reversible) type and of the heterotransfer (irreversible) type (Figure 9).

**Figure 10:**
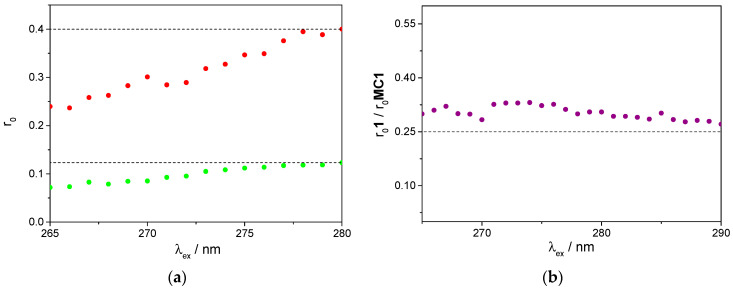
Fluorescence excitation anisotropy spectra of **1** (green) and **A** (red) in a Zeonex film at room temperature (**a**), and the respective anisotropy ratio (**b**), as a function of excitation wavelength. Slits were 8 nm for excitation and 14 nm for emission. $\lambda_{\mathrm{ex}}$ = 270 nm.

The dominant energy transfer mechanism is likely Förster resonance energy transfer (FRET) type. The computed Förster radii are 1.4 nm for homotransfer and 1.3 nm for heterotransfer. Given that average distances between neighboring chromophores are of the order of 0.5 to 0.7 nm for homotransfer, but probably somewhat higher for heterotransfer (conformation and structure dependent, between 0.6 and 1.0 nm), it is expected that efficient and fast (a few picoseconds, on average) energy hopping takes place in the calixarene skeleton, before the decay of the substituted phenoxy chromophores occurs either by the intrinsic radiative and nonradiative channels, or by irreversible FRET to the phthalimide group(s) (Figure 9). Evidence for homotransfer is obtained from fluorescence anisotropy measurements of both **1** (model compound for the calixarene chromophore) and **A** in a rigid medium (Zeonex film). The respective excitation fluorescence anisotropy spectra are shown in Figure 10, as well as the ratio of the two anisotropies.

The anisotropy *r*_0_(λ_exc_) of the model compound **A** (Figure 10) attains the value of 0.40 at the S_1_←S_0_ absorption onset (280 nm), corresponding to collinear absorption and emission transition dipole moments [38] (^1^L_b_ band), as expected. The excitation wavelength-dependence of the anisotropy, with a progressive decrease along the first band down to 0.24 at 260 nm, is compatible with a mixed polarisation, where the effect of the orthogonally-polarised ^1^L_a_ band increases when decreasing the excitation wavelength, as observed by Weber [39] for both *p*-cresol and tyrosine (but unlike the results reported in [40]). The fluorescence anisotropy excitation spectrum of parent **1** (Figure 10) follows the same trend, albeit with lower values, starting from 0.12 at 280 nm. The drop in anisotropy observed for this calixarene can be entirely ascribed to fast energy migration (FRET homotransfer). Given that there are four equivalent chromophores, with nearly isotropic and uncorrelated orientations, the anisotropy is expected to reduce to *r*_0_(λ_exc_)/4 owing to FRET [41]. As seen in Figure 10, this is indeed borne out by the experiment, with a ratio of 0.30 ± 0.03 being obtained between 265 and 290 nm. The slight excess over 0.25 can be ascribed to a residual degree of orientational anisotropy and orientational correlation. The fluorescence quantum yield decrease (by one or two orders of magnitude) in the case of compounds **2a**, **3a**, **3b** and **5a** results mainly from long-range quenching by the phthalimides owing to irreversible FRET. The two compounds bearing two acceptor phthalimides, **3a** and **3b**, display similar but lower donor fluorescence quantum yields when compared to the monophthalimide compound **2a**, as expected. The enhanced phthalimide emission in the **3b** case may result from the existence of two acceptors both in close proximity of the calixarene fluorophores and in a non self-quenching geometry. The monophthalimide **5a** differs from all the others by the number of carbon atoms of the spacer attaching the phthalimide (C_2_ vs. C_3_). The lower fluorescence quantum yield of **5a** (in fact, the lowest of all) is in accordance with the shortest (C_2_) chain, hence the shortest average donor-acceptor distance (down to 0.6 nm).

## 3. Material and Methods

### 3.1. Synthesis

All chemicals were reagent grade and were used without further purification. Microwave-assisted syntheses were performed with a CEM Discover SP microwave synthesizer system (2.45 GHz, 300 W) equipped with a non-contact infrared temperature sensor. The reaction temperature was controlled by variable microwave irradiation (0–150 W) and cooling by nitrogen current. The mechanosyntheses were performed on a PM100 Planetary Ball Mill (Retsch, Haan, Germany) using a 50 mL stainless steel or zirconium oxide reactor, and 200 balls with 5 mm of diameter each, of the corresponding materials. Chromatographic separations were performed on Merck silica gel 60 (particle size 40–63 μm, 230–400 mesh). Melting points were measured on a Stuart Scientific apparatus and are uncorrected. FTIR spectra were recorded on a Shimadzu Model IRaffinity-1 spectrophotometer (Kyoto, Japan). ^1^H and ^13^C NMR spectra were recorded on a Bruker Avance III 500 MHz spectrometer (Bruker, Billerica, MA, USA), with TMS as internal reference. The conventional COSY 45 and the phase-sensitive NOESY experiments were collected as 256 × 2 K complex points. Elemental analysis was determined on a Fisons EA 1108 microanalyser (Milano, Italy).

#### 3.1.1. Conventional Procedure for the Synthesis of **2a**, **3a** and **3b**

A mixture of *p-tert*-butyldihomooxacalix[4]arene (1 g, 1.47 mmol), *N*-(3-bromopropyl)phthalimide (0.81 g, 2.95 mmol) and K_2_CO_3_ (0.41 g, 2.95 mmol) in CH_3_CN (25 mL) was refluxed and stirred under N_2_ for 5 days. After cooling, the solvent was evaporated under reduced pressure and the residue was dissolved in CH_2_Cl_2_ (50 mL) and washed with 1 M HCl (2 × 30 mL), NH_4_Cl saturated solution (2 × 30 mL) and brine (30 mL). The organic layer was dried over Na_2_SO_4_, filtered and the solvent evaporated to dryness. The crude product was subjected to flash chromatography on silica gel (eluent CH_2_Cl_2_/MeOH, 99:1). According to TLC analysis, the fractions were combined into three sets, corresponding to derivatives **2a**, **3a** and **3b**, respectively.

7,13,19,25-Tetra-*tert*-butyl-28-[(3-phthalimidopropyl)oxy]-27,29,30-tri-hydroxy-2,3-dihomo-3-oxacalix[4]arene (**2a**): This compound was obtained in ≈ 2% yield (25 mg). An analytically pure sample was obtained by recrystallization from CH_2_Cl_2_/*n*-hexane. mp 171–173 °C; IR (KBr) 1717 cm^−1^ (CO), 3377 cm^−1^ (OH); ^1^H NMR (CDCl_3_, 500 MHz) *δ* 1.13, 1.230, 1.233, 1.26 [4s, 36H, C(CH_3_)], 2.45, 2.57 (2m, 2H, OCH_2_C*H*_2_CH_2_N), 3.31, 4.48 (ABq, 2H, *J* = 12.9 Hz, ArCH_2_Ar), 3.46, 4.31 (ABq, 2H, *J* = 13.8 Hz, ArCH_2_Ar), 3.54, 4.11 (ABq, 2H, *J* = 13.8 Hz, ArCH_2_Ar), 4.01, 4.17 (2m, 2H, OCH_2_CH_2_C*H*_2_N), 4.14–4.21 (m, 2H, OC*H*_2_CH_2_CH_2_N), 4.23, 4.94 (ABq, 2H, *J* = 9.3 Hz, CH_2_OCH_2_), 4.43, 4.70 (ABq, 2H, *J* = 10.0 Hz, CH_2_OCH_2_), 6.87, 6.95, 7.03, 7.07, 7.09, 7.13, 7.28, 7.30 (8d, 8H, ArH), 7.72, 7.87 (2m, 4H, ArH-Pht), 7.73, 8.47, 9.08 (3s, 3H, OH); ^13^C NMR (CDCl_3_, 125.8 MHz) *δ* 29.4 (OCH_2_*C*H_2_CH_2_N), 30.1, 31.3, 32.8 (ArCH_2_Ar), 31.1, 31.4, 31.5, 31.6 [C(*C*H_3_)_3_], 33.8, 33.9, 34.2 [*C*(CH_3_)_3_], 35.2 (OCH_2_CH_2_*C*H_2_N), 71.7, 72.2 (CH_2_OCH_2_), 74.2 (O*C*H_2_CH_2_CH_2_N), 122.6, 122.7, 126.5, 126.7, 127.4, 128.4, 131.3, 132.2, 132.8, 141.5, 142.4, 143.5, 147.8, 147.9, 149.3, 151.3, 152.7 (Ar), 123.4, 133.9 (ArH-Pht), 123.7, 125.2, 125.5, 125.6, 125.9, 126.7, 127.4, 128.1 (ArH), 168.3 (CO); Anal. Calcd for C_56_H_67_O_7_N: C, 77.66; H, 7.80; N, 1.62. Found: C, 77.18; H, 7.60; N, 1.38.

7,13,19,25-Tetra-*tert*-butyl-27,29-bis[(3-phthalimidopropyl)oxy]-28,30-di-hydroxy-2,3-dihomo-3-oxacalix[4]arene (**3a**): This compound was obtained in 54% yield (0.84 g). An analytically pure sample was obtained by recrystallization from CH_2_Cl_2_/MeOH. mp 172–174 °C; IR (KBr) 1709 cm^−1^ (CO), 3400 cm^−1^ (OH); ^1^H NMR (CDCl_3_, 500 MHz) *δ* 1.10, 1.19, 1.23, 1.24 [4s, 36H, C(CH_3_)], 2.55 (m, 4H, OCH_2_C*H*_2_CH_2_N), 3.25, 4.53 (ABq, 2H, *J* = 12.8 Hz, ArCH_2_Ar), 3.27, 4.67 (ABq, 2H, *J* = 12.8 Hz, ArCH_2_Ar), 3.47, 4.18 (ABq, 2H, *J* = 13.1 Hz, ArCH_2_Ar), 3.96, 4.13 (2m, 2H, OCH_2_CH_2_C*H*_2_N), 4.08, 4.43 (2m, 2H, OC*H*_2_CH_2_CH_2_N), 4.04–4.15 (2m, 4H, OC*H*_2_CH_2_C*H*_2_N), 4.23, 4.89 (ABq, 2H, *J* = 9.5 Hz, CH_2_OCH_2_), 4.37, 4.70 (ABq, 2H, *J* = 10.1 Hz, CH_2_OCH_2_), 6.81, 6.91, 6.96, 7.00, 7.04, 7.09, 7.23, 7.44 (8d, 8H, ArH), 7.20, 8.04 (2s, 2H, OH), 7.61, 7.68, 7.73, 7.83 (4m, 8H, ArH-Pht); ^13^C NMR (CDCl_3_, 125.8 MHz) *δ* 29.3, 30.0 (OCH_2_*C*H_2_CH_2_N), 29.8, 29.9, 32.9 (ArCH_2_Ar), 31.2, 31.4, 31.5, 31.6 [C(*C*H_3_)_3_], 33.8, 34.1 [*C*(CH_3_)_3_], 35.4, 35.7 (OCH_2_CH_2_*C*H_2_N), 71.9, 72.6 (CH_2_OCH_2_), 72.7, 74.2 (O*C*H_2_CH_2_CH_2_N), 122.2, 127.6, 128.0, 128.8, 129.2, 132.2, 132.3, 132.5, 132.6, 135.3, 141.2, 141.9, 146.4, 147.0, 149.4, 150.0, 152.8, 154.3 (Ar), 123.1, 123.2, 133.6, 133.8 (ArH-Pht), 123.7, 124.7, 125.1, 125.2, 125.6, 126.0, 127.7, 129.5 (ArH), 168.3, 168.4 (CO). Anal. Calcd for C_67_H_76_O_9_N_2_: C, 76.40; H, 7.27; N, 2.66. Found: C, 76.01; H, 7.63; N, 2.67.

7,13,19,25-Tetra-*tert*-butyl-28,29-bis[(3-phthalimidopropyl)oxy]-27,30-di-hydroxy-2,3-dihomo-3-oxacalix[4]arene (**3b**): This compound was obtained in 7% yield (0.11 g). An analytically pure sample was obtained by recrystallization from CH_2_Cl_2_/MeOH. mp 145–147 °C; IR (KBr) 1715 cm^−1^ (CO), 3397 cm^−1^ (OH); ^1^H NMR (CDCl_3_, 500 MHz) *δ* 1.06, 1.25 [2s, 36H, C(CH_3_)], 2.46, 2.52 (2m, 4H, OCH_2_C*H*_2_CH_2_N), 3.38, 4.29 (ABq, 4H, *J* = 13.7 Hz, ArCH_2_Ar), 3.39, 4.56 (ABq, 2H, *J* = 13.0 Hz, ArCH_2_Ar), 3.93–4.05 (m, 4H, OCH_2_CH_2_C*H*_2_N), 4.03, 4.25 (2m, 4H, OC*H*_2_CH_2_CH_2_N), 4.25, 4.71 (ABq, 4H, *J* = 9.7 Hz, CH_2_OCH_2_), 6.84, 6.90, 6.96, 7.21 (4d, 8H, ArH), 7.66, 7.78 (2m, 8H, ArH-Pht), 7.68 (s, 2H, OH); ^13^C NMR (CDCl_3_, 125.8 MHz) *δ* 29.4 (OCH_2_*C*H_2_CH_2_N), 30.8, 31.6 (ArCH_2_Ar), 31.2, 31.6 [C(*C*H_3_)_3_], 33.8, 34.0 [*C*(CH_3_)_3_], 35.4 (OCH_2_CH_2_*C*H_2_N), 71.7 (CH_2_OCH_2_), 73.1 (O*C*H_2_CH_2_CH_2_N), 123.17, 133.8 (ArH-Pht), 123.22, 128.2, 132.2, 132.6, 133.4, 141.7, 146.4, 151.5, 152.1 (Ar), 124.7, 125.2, 125.8, 128.0 (ArH), 168.3 (CO); Anal. Calcd for C_67_H_76_O_9_N_2_: C, 76.40; H, 7.27; N, 2.66. Found: C, 76.34; H, 7.62; N, 2.63.

#### 3.1.2. Conventional Procedure for the Synthesis of **5a** and **6a**

A mixture of **1** (1 g, 1.47 mmol) and K_2_CO_3_ (0.81 g, 5.9 mmol) in MeCN (25 mL) was stirred and gently warmed under N_2_ for 30 min. After cooling, *N*-(2-bromoethyl)-phthalimide (1.58 g, 5.9 mmol) and KI (0.49 g, 2.95 mmol) were added, and the reaction mixture was refluxed and stirred under N_2_ for 3 days. By the same workup described above, the crude product obtained was subjected to flash chromatography on silica gel (eluent gradient from *n*-hexane/ethyl acetate, 90:10 to 70:30). According to TLC analysis, the fractions were combined into two sets, corresponding mainly to derivatives **5a** and **6a**, respectively.

7,13,19,25-Tetra-*tert*-butyl-28-[(2-phthalimidoethyl)oxy]-27,29,30-tri-hydroxy-2,3-dihomo-3-oxacalix[4]arene (**5a**): This compound was obtained in 24% yield (0.30 g). An analytically pure sample was obtained by recrystallization from CH_2_Cl_2_/MeOH. mp 176–178 °C; IR (KBr) 1717 cm^−1^ (CO), 3375 cm^−1^ (OH); ^1^H NMR (CDCl_3_, 500 MHz) *δ* 1.11, 1.21, 1.222, 1.224 [4s, 36H, C(CH_3_)], 3.24, 4.25 (ABq, 2H, *J* = 12.8 Hz, ArCH_2_Ar), 3.38, 4.15 (ABq, 2H, *J* = 13.7 Hz, ArCH_2_Ar), 3.52, 4.09 (ABq, 2H, *J* = 13.6 Hz, ArCH_2_Ar), 4.20, 4.86 (ABq, 2H, *J* = 9.1 Hz, CH_2_OCH_2_), 4.28, 4.40 (ABq, 2H, *J* = 10.0 Hz, CH_2_OCH_2_), 4.29, 4.49 (2m, 2H, OC*H*_2_CH_2_N), 4.39 (t, 2H, OCH_2_C*H*_2_N), 6.78, 6.94, 6.99, 7.06, 7.07, 7.08, 7.19, 7.26 (8d, 8H, ArH), 7.28, 8.25, 8.60 (3s, 3H, OH), 7.68, 7.91 (2m, 4H, ArH-Pht); ^13^C NMR (CDCl_3_, 125.8 MHz) *δ* 29.8, 31.2, 32.2 (ArCH_2_Ar), 31.1, 31.4, 31.5, 31.6 [C(*C*H_3_)_3_], 33.77, 33.81, 33.9, 34.2 [*C*(CH_3_)_3_], 37.5 (OCH_2_*C*H_2_N), 71.6, 71.9 (CH_2_OCH_2_), 72.0 (O*C*H_2_CH_2_N), 122.2, 122.7, 126.4, 126.7, 127.3, 127.9, 131.5, 132.5, 132.5, 141.2, 142.2, 143.3, 147.8, 147.9, 148.6, 151.3, 152.5 (Ar), 123.4, 133.7 (ArH-Pht), 123.7, 125.2, 125.3, 125.5, 125.7, 126.7, 127.3, 128.2 (ArH), 168.5 (CO); Anal. Calcd for C_55_H_65_O_7_N: C, 77.52; H, 7.69; N, 1.64. Found: C, 77.12; H, 8.01; N, 1.65. Although this compound had been synthesised before by a slightly different procedure [34], neither the mp nor the ^1^H and ^13^C NMR chemical shifts are totally correct.

7,13,19,25-Tetra-*tert*-butyl-27,29-bis[(3-phthalimidoethyl)oxy]-28,30-di-hydroxy-2,3-dihomo-3-oxacalix[4]arene (**6a**): This compound was obtained in 18% yield (0.27 g). ^1^H NMR (CDCl_3_, 500 MHz) *δ* 1.06, 1.20, 1.21, 1.25 [4s, 36H, C(CH_3_)], 3.17, 4.37 (ABq, 2H, *J* = 13.0 Hz, ArCH_2_Ar), 3.30, 4.67 (ABq, 2H, *J* = 13.0 Hz, ArCH_2_Ar), 3.48, 4.18 (ABq, 2H, *J* = 13.3 Hz, ArCH_2_Ar), 4.21, 4.92 (ABq, 2H, *J* = 9.5 Hz, CH_2_OCH_2_), 4.25–4.34 (m, 2H, OCH_2_C*H*_2_N),4.32, 4.77 (2m, 2H, OC*H*_2_CH_2_N), 4.38, 4.74 (ABq, 2H, *J* = 10.3 Hz, CH_2_OCH_2_), 4.42, 4.55 (2m, 2H, OC*H*_2_CH_2_N), 4.51 (m, 2H, OCH_2_C*H*_2_N), 6.78, 6.82, 6.95, 7.00, 7.09, 7.16, 7.41 (7d, 8H, ArH), 7.14, 7.75 (2s, 2H, OH), 7.61, 7.69, 7.82, 7.85 (4m, 8H, ArH-Pht); ^13^C NMR (CDCl_3_, 125.8 MHz) *δ* 29.8, 30.0, 32.7 (ArCH_2_Ar), 31.1, 31.4, 31.5, 31.6 [C(*C*H_3_)_3_], 33.77, 33.79, 34.1 [*C*(CH_3_)_3_], 37.4, 38.4 (OCH_2_*C*H_2_N), 70.1, 71.7, 72.8 (CH_2_OCH_2_ and O*C*H_2_CH_2_N), 122.0, 127.4, 127.8, 128.6, 129.0, 132.1, 132.3, 132.4, 134.7, 141.0, 141.9, 146.4, 147.1, 149.5, 149.7, 152.5, 154.0 (Ar), 123.1, 123.3, 133.6, 133.9 (ArH-Pht), 123.3, 124.8, 125.2, 125.3, 125.6, 126.0, 127.5, 129.6 (ArH), 168.1, 168.5 (CO).

#### 3.1.3. Microwave- and Mechanically-Assisted Syntheses

MW procedure for the synthesis of **2a**, **3a** and **3b**: A mixture of **1** (0.30 g, 0.44 mmol), *N*-(3-bromopropyl)phthalimide (0.24 g, 0.89 mmol) and K_2_CO_3_ (0.12 g, 0.89 mmol) was suspended in CH_3_CN (3 mL) and irradiated under stirring for 30 min at 160 °C. The products were isolated and purified by the same workup and purification steps described in 3.1.1.

MW procedure for the synthesis of **6a**: A mixture of **1** (0.30 g, 0.44 mmol) and K_2_CO_3_ (0.24 g, 1.77 mmol) was suspended in CH_3_CN (3 mL) and irradiated under stirring for 6 min at 110 °C. After cooling, *N*-(2-bromoethyl)phthalimide (0.45 g, 1.77 mmol) was added and the reaction mixture irradiated for a further 36 min at 150 °C. The product was obtained by the same steps described in 3.1.2.

Mechanochemical reaction protocol: 50 mg of parent **1**, *N*-(bromoalkyl)phthalimide and K_2_CO_3_ were loaded into the selected reactor containing the corresponding 200 balls. The reactor was then adjusted into the planetary ball mill and the mixture was grinded at 500 rpm, with rotation inversion cycles of 30 min (2.5 min. pause between inversion cycles). Different reagent ratios and reaction times were tested, as mentioned before (see 2.1.). The products were obtained by the same workup described previously.

### 3.2. Determination of the Crystallographic Structures of **2a**, **3a**, **3b** and **5a**

Single crystals suitable for X-ray investigation were obtained by slow evaporation of acetonitrile solutions containing compound **2a**, **3a** or **3b**, while dichloromethane/ethanol solvent mixtures were used for **5a**. Data collection was carried out at the XRD1 beamline of the Elettra synchrotron (Trieste, Italy), employing the rotating-crystal method with a Dectris Pilatus 2M area detector. Single crystals were dipped in paratone cryoprotectant, mounted on a loop and flash-frozen under a nitrogen stream at 100 K. Diffraction data were indexed and integrated using the XDS package [42], while scaling was carried out with XSCALE [43]. Structures were solved using the SHELXT program [44] and the refinement was performed with SHELXL-14 [45], operating through the WinGX GUI [46], by full-matrix least-squares (FMLS) methods on F^2^. Non-hydrogen atoms were anisotropically refined with the exception of non-hydrogen atoms with a low occupancy factor, which were refined isotropically. Hydrogen atoms, located on the difference Fourier maps, were added at the calculated positions and refined using the riding model. Crystallographic data and refinement details are reported in Table S4.

### 3.3. Fluorescence Studies

Absorption and fluorescence studies were performed using an UV-3101PC UV-Vis-NIR spectrophotometer (Shimadzu, Kyoto, Japan) and a Fluorolog F112A fluorimeter (Spex Industries, Edison, NJ, USA) in right-angle configuration, respectively. The absorption spectra were recorded between 240 and 340 nm and the emission ones between 285 and 600 nm, and using quartz cells with an optical path length of 1 cm. Details concerning the photophysical properties determination are given in the Supplementary Materials.

## 4. Conclusions

The alkylation of *p-tert*-butyldihomooxacalix[4]arene (**1**) with *N*-(bromopropyl)- or *N*-(bromoethyl)phthalimides and K_2_CO_3_ in MeCN was carried out by conventional heating and by microwave irradiation and ball milling methodologies. Eight compounds were identified and five isolated (**2a**, **3a**, **3b**, **5a** and **6a**), including mono- and mainly 1,3-disubstituted derivatives in the cone conformation. A microwave-assisted procedure was developed, affording both 1,3-disubstituted phthalimides (**3a** and **6a**) as major/unique products. The reaction times were drastically reduced (30–42 min) and the selectivity increased when compared to refluxing conditions (several days for completion). Minor amounts of solvent and less reactants used were other advantages of this environmentally friendly technique. The results obtained open new perspectives for using this MW-assisted protocol in the synthesis of new calixarenes. Mechanically-assisted synthesis was not shown to be a good method for these alkylation reactions. The conformation and the substitution patterns were confirmed by NMR spectroscopy (^1^H, ^13^C, COSY and NOESY) and X-ray diffraction. The X-ray structural analysis of **2a**, **3a**, **3b** and **5a** reveals a common open conformation of the calixarene cone, determined by an intramolecular H-bond which involves the oxygen acceptor of the oxa bridge oriented towards the center of the macrocycle. The lower rim substitution patterns, which determine the intramolecular H-bond network, strongly influence the orientation and π-stacking interactions of the phthalimide arms. The photophysical properties of **2a**, **3a**, **3b** and **5a** were also determined. In particular, it is found that upon excitation of the arene fluorophores, fast energy migration (FRET homotransfer) within the ring ensues, followed by irreversible energy transfer (FRET heterotransfer) to the phthalimide chromophores. These phthalimide derivatives will be used as precursors to urea-terminated anion-binding macrocycles. The ureido-dihomooxacalix[4]arenes bearing propyl or ethyl spacers are expected to be better receptors than those with butyl spacers, mainly for ion pairs, as they will be in a closer proximity. This study is currently under investigation.

## Figures and Tables

**Table 1:** Conditions and product percentages of reaction of **1** with Br(CH_2_)_3_Pht.

Entry	Method	Reaction Time	1:RX:Base Ratio	Reaction Mixture (%) ^1^				
				1	2a	3a	3b	4
1	Reflux	4 days	1:2:2	6	13	65	3	12
2	Reflux	5 days	1:2:2	—	4	76	15	5
3	MW	20 min	1:2:2	—	10	86	2	2
4	MW	30 min	1:2:2	—	3	93	2	2
5	BM	2 h	1:2:2	70	30	—	—	—
6	BM	12 h	1:4:4	27	46	19	4	4

^1^ Percentage of product formation in the reaction mixture determined by ^1^H NMR.

**Table 2:** Conditions and product percentages of reaction of **1** with Br(CH_2_)_2_Pht.

Entry	Method	Reaction Time	1:RX:Base:KI Ratio	Reaction Mixture (%) ^1^				
				1	5a	5b	6a	6b
1	Reflux	3 days	1:2:2:0	61	21	6	11	1
2	Reflux	3 days	1:2.5:2:2	30	45	7	16	2
3	Reflux	3 days	1:4:4:2	19	44	4	30	3
4	Reflux	7 days	1:6:6:4	18	37	12	30	3
5	MW	6 + 12 min	1:4:2:2	16	58	—	26	—
6	MW	6 + 36 min	1:4:2:2	—	59	—	41	—
7	MW	12 + 36 min	1:4:2:2	—	38	—	62	—
8	MW	12 + 36 min	1:4:2:0	—	43	—	57	—
9	MW	18 + 36 min	1:4:4:0	—	—	—	100	—
10	MW	6 + 36 min	1:4:4:0	—	—	—	100	—
11	BM	12 h	1:4:4:0	82	18	—	—	—

^1^ Percentage of product formation in the reaction mixture determined by ^1^H NMR.

**Table 3:** Comparison of cone conformations: Dihedral angles (canting angles) between corresponding aryl planes of the calixarene cones (A, B, C and D) and the mean planes of the bridging methylene carbon atoms for various dihomooxacalix[4]arenes.

Molecule		A	B	C	D
**2a** *	(I)	138.4	154.7	105.0	126.1
	(II)	141.0	146.1	107.9	132.5
	(III)	138.6	149.5	102.3	122.4
	(IV)	128.0	148.8	98.5	129.9
**3a** **	(I)	137.02	145.72	100.30	118.38
	(II)	134.54	146.88	100.23	118.73
	(III)	135.17	147.47	101.34	116.39
**3b**		140.96	146.90	94.82	120.85
**5a**		144.25	149.98	106.82	118.39

* Four crystallographically independent molecules in the asymmetric unit; ** Three crystallographically independent molecules in the asymmetric unit. See Figure 5 for description of aryl rings A, B, C and D.

**Table 4:** Photophysical properties of the studied compounds in CH_2_Cl_2_ at 293 K.

	*λ*_max,abs_ (nm)	*ε* (M^–1^ cm^–1^)	*λ*_max,em_ (nm)	Stokes Shift ^a^ (nm)	*τ*_f_ (ns)	*Φ*_F_^b^
**A**	271	5.0 × 10^2^	288	17	4.50 ^c^	7.5 × 10^−3^
**B**	294	8.2 × 10^2^	403	109	0.26 ^d^	1.8 × 10^−3^
**1**	286	3.6 × 10^4^	306	20	1.50 ^c^	1.3 × 10^−1^
**2a**	288	2.0 × 10^4^	310	22	—	1.1 × 10^−2^
**3a**	290	1.9 × 10^4^	314	22	1.32 ^d^	1.7 × 10^−3^
**3b**	282	1.7 × 10^4^	314	30	3.57 ^d^	2.5 × 10^−3 e^
**5a**	288	2.9 × 10^4^	310	22	—	1.4 × 10^−3^

^a^ Computed as $\lambda_{\max,\mathrm{em}}-\lambda_{\max,\mathrm{abs}}$; ^b^ Against quinine sulfate *Φ*_F_ = 0.546 in H_2_SO_4_ 0.5 M; ^c^$\;\lambda_{\mathrm{ex}}$ = 280 nm and $\lambda_{\mathrm{em}}$ = 306 nm; ^d^ $\lambda_{\mathrm{ex}}$ = 295 nm and $\lambda_{\mathrm{em}}$ = 400 nm; ^e^ After subtraction of the fluorescence of the phthalimide moiety (peaking at 400 nm, see Figure 8c).

## Data Availability

Not applicable.

## Supplementary Materials

The following are available online: ^1^H NMR assignments of compounds **2b**, **5b** and **6b**; Crystallographic data and refinement details; Photophysical properties determination; ^1^H, ^13^C, COSY and NOESY spectra of compounds **2a**, **3a**, **3b**, **5a** and **6a**.

## Sample Availability

Samples of the compounds are not available from the authors.

## References

[1] Gutsche C.D. (2008). Calixarenes, an Introduction.

[2] Neri P., Sessler J.L., Wang M.-X. (2016). Calixarenes and Beyond.

[3] Busschaert N., Caltagirone C., Van Rossom W., Gale P.A. (2015). Applications of supramolecular anion recognition. Chem. Rev..

[4] Gale P.A., Howe E.N.W., Wu X. (2016). Anion receptor chemistry. Chem.

[5] Gedye R., Smith F., Westaway K., Ali H., Baldisera L., Laberge L., Rousell J. (1986). The use of microwave ovens for rapid organic synthesis. Tetrahedron Lett..

[6] Giguere R.J., Bray T.L., Duncan S.M. (1986). Application of commercial microwave ovens to organic synthesis. Tetrahedron Lett..

[7] Strauss C.R. (2009). A strategic, “green” approach to organic chemistry with microwave assistance and predictive yield optimization as core, enabling technologies. Aust. J. Chem..

[8] Caddick S., Fitzmaurice R. (2009). Microwave enhanced synthesis. Tetrahedron.

[9] Gawande M.B., Shelke S.N., Zboril R., Varma R.S. (2014). Microwave-assisted chemistry: Synthetic applications for rapid assembly of nanomaterials and organics. Acc. Chem. Res..

[10] Kumar A., Kuang Y., Liang Z., Sun X. (2020). Microwave chemistry, recent advancements, and eco-friendly microwave-assisted synthesis of nanoarchitectures and their applications: A review. Mater. Today Nano.

[11] Stolle A., Szuppa T., Leonhardt S.E.S., Ondruschka B. (2011). Ball milling in organic synthesis: Solutions and challenges. Chem. Soc. Rev..

[12] James S.L., Adams C.J., Bolm C., Braga D., Collier P., Friscie T., Grepioni F., Harris K.D.M., Hyett G., Jones W. (2012). Mechanochemistry: Opportunities for new and cleaner synthesis. Chem. Soc. Rev..

[13] Wang G.W. (2013). Mechanochemical organic synthesis. Chem. Soc. Rev..

[14] Rahman M., Santra S., Kovalev I.S., Kopchuk D.S., Zyryanov G.V., Majee A., Chupakhin O.N. (2020). Gree synthetic approaches for practically relevant (hetero)macrocycles: An overview. AIP Conf. Proc..

[15] Baozhi L., Gengliang Y., Jinsong Z., Kefang D. (2005). Microwave-assisted synthesis of *p*-alkylcalix[n]arene catalysed by KOH. Eur. J. Chem..

[16] Takagaki M., Hosoda A., Mori H., Miyake Y., Kimura K., Taniguchi H., Nomura E. (2008). Rapid and convenient laboratory method for the preparation of *p-tert*-butylcalix[4]arene using microwave irradiation. Green Chem..

[17] Agrawal Y.K., Desai N.C., Mehta N.D. (2007). Microwave-assisted synthesis of azocalixarenes. Synth. Commun..

[18] Cecioni S., Lalor R., Blanchard B., Praly J.P., Imberty A., Matthews S.E., Vidal S. (2009). Achieving high affinity towards a bacterial lectin through multivalent topological isomers of calix[4]arene glycoconjugates. Chem. Eur. J..

[19] Fatykhova G.A., Burilov V.A., Dokuchaeva M.N., Solov’eva S.E., Antipin I.S. (2018). Synthesis of tetraazide derivatives of *p-tert*-butylcalix[4]arene using copper-catalyzed nucleophilic aromatic substitution. Dokl. Chem..

[20] Wang X., Zhao Z., Chen B., Li X., Liu M. (2015). Microwave-assisted synthesis of bidentate chiral unsymmetrical urea derivatives of *p-tert*-butylcalix[4]arene and their anion recognition properties. J. Chem. Res..

[21] Nayak S.K., Choudhary M.K. (2012). Microwave-assisted synthesis of 1,3-dialkyl ethers of calix[4]arenes: Application to the synthesis of cesium selective calix[4]crown-6 ionophores. Tetrahedron Lett..

[22] Burilov V.A., Nugmanov R.I., Ibragimova R.R., Solovieva S.E., Antipin I.S., Konovalov A.I. (2013). Microwave-assisted alkylation of *p-tert*-butylcalix[4]arene lower rim: The effect of alkyl halides. Mendeleev Commun..

[23] Bakic M.T., Klaric D., Espinosa M.S., Kazazic S., Frkanec L., Babay P.A., Galic N. (2019). Syntheses of ester and amide derivatives of calix[6]arene and their complexation affinities towards La^3+^, Eu^3+^, and Yb^3+^. Supramol. Chem..

[24] Galán H., de Mendoza J., Prados P. (2010). Microwave-assisted synthesis of a nitro-*m*-xylylenedioxycalix[6]arene building block functionalized at the upper rim. Eur. J. Org. Chem..

[25] Atwood J.L., Hardie M.J., Raston C.L., Sandoval C.A. (1999). Convergent synthesis of *p*-benzylcalix[7]arene: Condensation and UHIG of *p*-benzylcalix[6 or 8]arenes. Org. Lett..

[26] Chennakesavulu K., Raju G.B. (2010). Mechanochemical synthesis of *p*-nitro calix[6]arene. Asian J. Chem..

[27] Marcos P.M., Teixeira F.A., Segurado M.A.P., Ascenso J.R., Bernardino R.J., Michel S., Hubscher-Bruder V. (2014). Bidentate urea derivatives of *p-tert*-butyldihomooxacalix[4]arene: Neutral receptors for anion complexation. J. Org. Chem..

[28] Marcos P.M., Teixeira F.A., Segurado M.A.P., Ascenso J.R., Bernardino R.J., Brancatelli G., Geremia S. (2014). Synthesis and anion binding properties of new dihomooxacalix[4]arene diurea and dithiourea receptors. Tetrahedron.

[29] Augusto A.S., Miranda A.S., Ascenso J.R., Miranda M.Q., Félix V., Brancatelli G., Hickey N., Geremia S., Marcos P.M. (2018). Anion recognition by partial cone dihomooxacalix[4]arene-based receptors bearing urea groups: Remarkable affinity for benzoate ion. Eur. J. Org. Chem..

[30] Miranda A.S., Serbetci D., Marcos P.M., Ascenso J.R., Berberan-Santos M.N., Hickey N., Geremia S. (2019). Ditopic receptors based on dihomooxacalix[4]arenes bearing phenylurea moieties with electron-withdrawing groups for anions and organic ion pairs. Front. Chem..

[31] Miranda A.S., Marcos P.M., Ascenso J.R., Berberan-Santos M.N., Schurhammer R., Hickey N., Geremia S. (2020). Dihomooxacalix[4]arene-based fluorescent receptors for anion and organic ion pair recognition. Molecules.

[32] Barboso S., Carrera A.G., Matthews S.E., Arnaud-Neu F., Bohmer V., Dozol J.F., Rouquette H., Schiwing-Weill M.J. (1999). Calix[4]arenes with CMPO functions at the lower rim. Synthesis and extraction properties. J. Chem. Soc. Perkin Trans. 2.

[33] Marcos P.M., Ascenso J.R., Pereira J.L.C. (2002). Synthesis and NMR conformational studies of p-tert-butyldihomooxacalix[4]arene derivatives bearing pyridyl pendant groups at the lower rim. Eur. J. Org. Chem..

[34] Jaime C., de Mendoza J., Prados P., Nieto P., Sanchez C. (1991). Carbon-13 NMR chemical shifts. A single rule to determine the conformation of calix[4]arenes. J. Org. Chem..

[35] Liu Y., Sun J., Yan C.G. (2017). Synthesis and crystal structures of *p-tert*-butyldihomooxa-calix[4]arene mono-Schiff bases. J. Incl. Phenom. Macrocycl. Chem..

[36] Miranda A.S., Martelo L.M., Fedorov A.A., Berberan-Santos M.N., Marcos P.M. (2017). Fluorescence properties of *p-tert*-butyldihomooxacalix[4]arene derivatives and the effect of anion complexation. New J. Chem..

[37] Griesbeck A.G., Görner H. (1999). Laser flash photolysis study of *N*-alkylated phathalimides. J. Photochem. Photobiol..

[38] Valeur B., Berberan-Santos M.N. (2012). Molecular Fluorescence. Principles and Applications.

[39] Weber G. (1960). Fluorescence-polarization spectrum and electronic-energy transfer in tyrosine, tryptophan and related compounds. Biochem. J..

[40] Fornander L.H., Feng B., Beke-Somfai T., Nordén B. (2014). UV transition moments of tyrosine. J. Phys. Chem. B.

[41] Berberan-Santos M.N., Canceill J., Brochon J.C., Jullien L., Lehn J.-M., Pouget J., Tauc P., Valeur B. (1992). Multichromophoric cyclodextrins. 1. Synthesis of *O*-naphthoyl beta cyclodextrins and investigation of excimer formation and energy hopping. J. Am. Chem. Soc..

[42] Kabsch W. (2010). XDS. Acta Crystallogr. Sect. D Biol. Crystallogr..

[43] Kabsch W. (2010). Integration, scaling, space-group assignment and post-refinement. Acta Crystallogr. Sect. D Biol. Crystallogr..

[44] Sheldrick G.M. (2015). SHELXT–integrated space-group and crystal-structure determination. Acta Crystallog. Sect. A Found. Crystallogr..

[45] Sheldrick G.M. (2008). A short history of SHELX. Acta Crystallog. Sect. A Found. Crystallogr..

[46] Farrugia L.J. (2012). WinGX and ORTEP for windows: An update. J. Appl. Crystallog..

