# Supplementary material for: Conventional vs. Microwave- or Mechanically-Assisted Synthesis of Dihomooxacalix[4]arene Phthalimides: NMR, X-ray and Photophysical Analysis §
Source: Molecules. 2021 Mar 10;26(6):1503. doi: 10.3390/molecules26061503 (PMC7998390; doi:10.3390/molecules26061503)
Supplement: Supplementary file 1 [file molecules-26-01503-s001.pdf]

Supplementary Materials for

Conventional *vs* Microwave- or Mechanically-Assisted Synthesis of Dihomooxacalix[4]arene Phthalimides: NMR, X-ray and Photophysical Analysis

Alexandre S. Miranda,[1,2] Paula M. Marcos,[1,3,*] José R. Ascenso,[4] M. Paula Robalo,[4,5] Vasco D. B.Bonifácio,[2] Mário N. Berberan-Santos,[2] Neal Hickey[6] and Silvano Geremia[6]

List of contents

1. ^{1}H NMR assignments of compounds **2b**, **5b** and **6b** 2
2. Dihedral angles and H-bond interactions for various dihomooxacalix[4]arenes 4
3. Photophysical properties determination 6
4. Crystallographic data and refinement details 8
5. ^{1}H NMR spectra of **2a**, **3a**, **3b**, **5a** and **6a** 11
6. ^{13}C NMR spectra of **2a**, **3a**, **3b**, **5a** and **6a** 16
7. COSY spectra of **2a**, **3a**, **3b**, **5a** and **6a** 21
8. NOESY spectra of **2a**, **3a**, **3b**, **5a** and **6a** **26**

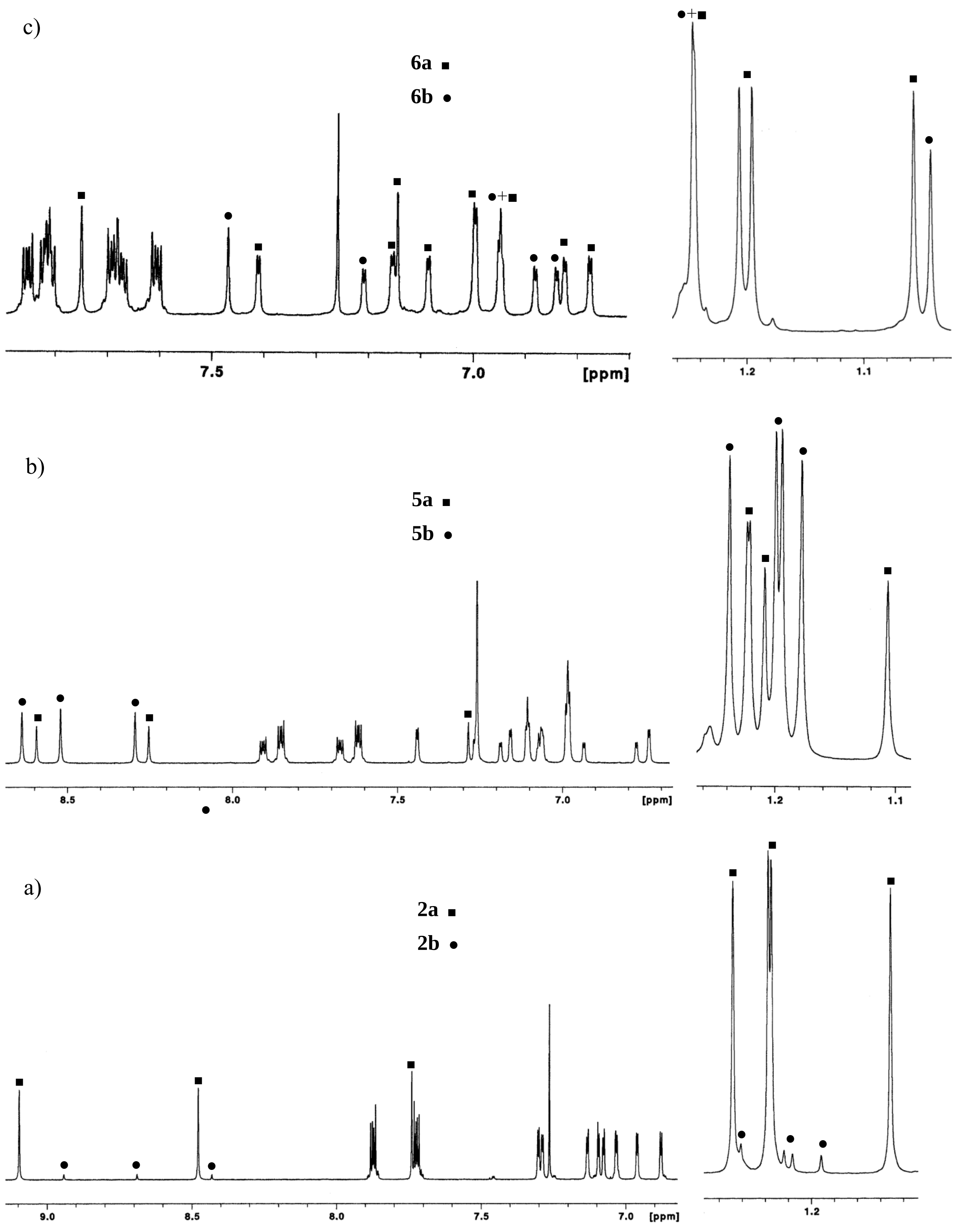

Figure S1. Partial ^{1}H NMR spectra (500 MHz, $CDCl_3$, 22 ℃) of compounds: (a) mono-propylphthalimides **2a**+**2b**, (b) mono-ethylphthalimides **5a**+**5b** and (c) 1,3-di- and 3,4-di-ethylphthalimides **6a**+**6b**.

Table S1a. ^{1}H NMR data (500 MHz, $CDCl_3$, 22 ℃) of dihomooxa phthalimide derivatives

Compd	OH_1	OH_2	OH_3	*t*-Bu_1	*t*-Bu_2	*t*-Bu_3	*t*-Bu_4
mono **2b**	8.43	8.68	8.94	1.19	1.21	1.22	1.25
mono **5b**	8.30	8.52	8.64	1.18	1.19	1.20	1.24
3,4-di **6b**	7.47	—	—	1.04	1.25	—	—

Table S1b. ^{1}H NMR data (500 MHz, $CDCl_3$, 22 ℃) of dihomooxa phthalimide derivatives

Compd	ArH_1	ArH_2	ArH_3	ArH_4	ArH_5	ArH_6	ArH_7	ArH_8
mono **5b**	6.74	6.98	6.99	6.99	7.10	7.11	7.16	7.44
3,4-di **6b**	6.84	6.88	6.95	7.21	—	—	—	—

Table S2. Comparison of intramolecular hydrogen bonding interactions found in various dihomooxacalix[4]arenes. H-bond distances of bifurcated H-bonds, with one donor and two acceptor atoms, are reported on the same row. Atom labels: O1A(B)(C)(D) = calixarene hydroxyl/alkoxyl O atom; O2B bridging O atom; O3C phthalimide O atom

molecule	D-H•••A	d(D•••A) (Å)
**2a*** (I)	O(1A)-H•••O(1D) •••O(3C)	2.885(7), 3.047(6)
	O(1B)-H•••O(1A) •••O(2B)	2.912(6), 2.789(6)
	O(1D)-H•••O(1C) •••O(3C)	2.682(6), 3.219(6)
(II)	O(1A)-H•••O(1D) •••O(3C)	2.775(6), 3.271(6)
	O(1B)-H•••O(1A) •••O(2B)	2.923(5), 2.862(6)
	O(1D)-H•••O(1C) •••O(3C)	2.692(5), 3.297(6)
(III)	O(1A)-H•••O(1D) •••O(3C)	2.884(6), 3.587(6)
	O(1B)-H•••O(1A) •••O(2B)	2.917(5), 2.784(5)
	O(1D)-H•••O(1C) •••O(3C)	2.734(5), 3.424(6)
(IV)	O(1A)-H•••O(1D) •••O(3C)	2.889(6), 3.237(19)
	O(1B)-H•••O(1A) •••O(2B)	3.088(5), 2.723(5)
	O(1D)-H•••O(1C) •••O(3C)	2.720(5), 3.040(19)
**3a****(I)	O(1B)-H•••O(1A) •••O(2B)	2.935(1), 2.776(1)
	O(1D)-H•••O(1C)	2.828(1)
(II)	O(1B)-H•••O(1A) •••O(2B)	2.900(1), 2.830(1)
	O(1D)-H•••O(1C)	2.813(1)
(III)	O(1B)-H•••O(1A) •••O(2B)	2.936(1), 2.792(1)
	O(1D)-H•••O(1C)	2.808(1)
**3b**	O(1A)-H•••O(1D)	2.793(1)
	O(1B)-H•••O(1A) •••O(2B)	3.129(1), 2.737(1)
**5a**	O(1A)-H•••O(1D) •••O(3C)	2.876(2), 3.133(2)
	O(1B)-H•••O(1A) •••O(2B)	2.851(2), 2.797(2)
	O(1D)-H•••O(1C) •••O(3C)	2.681(2), 2.974(2)

*Four crystallographically independent molecules; **Three crystallographically independent molecules.

Table S3. Comparison of phthalimide substituent orientation: Dihedral angles between the phthalimide planes and the mean plane of the bridging methylene carbon atoms (α plane), between the phthalimide planes and the corresponding aryl plane of the calixarene to which the substituent is attached (β plane) and between the two phthalimide planes (γ plane) are reported for various dihomoaxacalix[4]arenes. See Figure 5 for description of aryl rings A, B, C and D. The torsion angles of the phthalimide linker chain starting from the alkoxy bond are also reported.

molecule	aryl	α	β	γ	Torsion angles C-C-O-C-C-C-N-C				
**2a*** (I)	C	78.3	65.2		93.7	-176.9	-56.5	174.9	90.6
(II)	C	76.1	59.4		95.4	-177.5	-59.9	173.5	97.0
(III)	C	74.6	55.1		100.9	173.1	-65.0	169.1	104.7
(IV)	C	79.4	58.0		93.1	-173.6	-51.1	172.2	92.3
**3a****(I)	A	11.9	46.2	76.0	-82.6	-158.4	-175.8	-57.0	102.9
	C	83.3	33.0		-86.5	146.5	55.5	-179.0	76.4
(II)	A	42.7	26.8	15.8	-85.2	-174.0	-175.3	174.5	107.0
	C	28.1	76.5		-88.3	-165.4	-62.9	-62.7	135.3
(III)	A	9.4	48.3	87.9	-72.1	-137.7	172.3	-68.4	104.3
	C	85.1	61.0		-92.0	173.9	-64.2	-173.4	93.1
**3b**	C	32.9	113.3	8.6	-69.0	-92.4	-72.8	170.5	80.6
	D	24.4	54.4		-91.6	-175.7	168.1	57.4	82.8
**5a**	C	22.5	102.1		-87.8	166.3	66.9	2.0	#

*Four crystallographically independent molecules; **Three crystallographically independent molecules.
Torsion angles C-C-O-C-C-N-C.

Photophysical Properties Determination

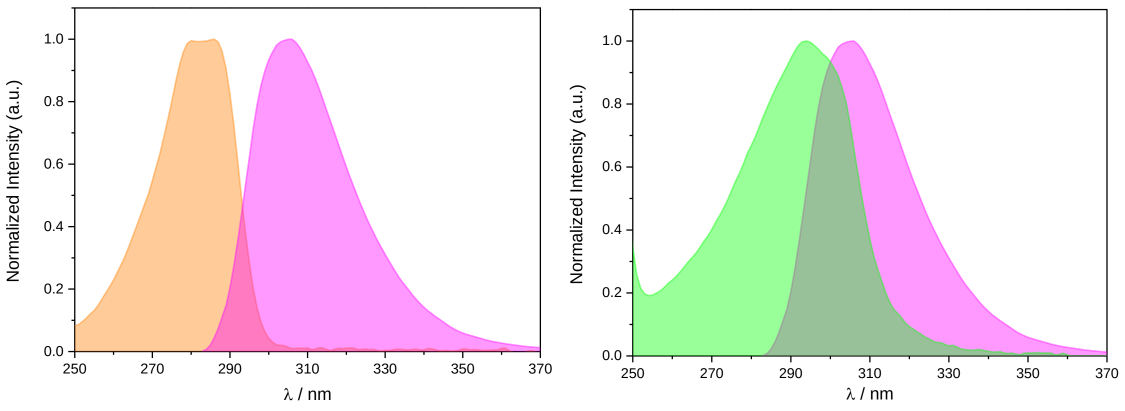

Figure S2. Absorption and fluorescence spectral overlap for **1-1** (homotransfer) (a) and for **1-B** (heterotransfer) (b) energy transfer donor-acceptor pairs.

Emission spectra were corrected for the spectral response of the optics and the photomultiplier. Fluorescence quantum yields were measured against quinine sulfate as (Φ_F = 0.546 in H_2SO_4 0.5 M) [1]. Fluorescence quantum yields were determined using equation 1:

$$\Phi = \Phi_r \left(\frac{I}{I_r}\right)\left(\frac{A_r}{A}\right)\left(\frac{n^2}{n_r^2}\right) \quad (1)$$

where Φ is the quantum yield, *I* is the integrated fluorescence emission intensity, *A* denotes the absorbance at the excitation wavelength, with subscript *r* referring to the reference, and *n* and n_r are the refractive indices of dichloromethane and water, respectively [2]. Time-resolved fluorescence intensity decays were obtained using the single-photon timing method with laser excitation and microchannel plate detection, with the set-up already described [3]. The excitation wavelength used was between 280 nm (**A** and **1**) and 295 nm (**B**, **3a** and **3b**) and the emission wavelengths was at the maximum emission for **A** and for **1** and 400 nm for **3a** and **3b**, using a front-face geometry. The timescale varied between 1.22 (**3a**) and 16.3 ps (**3b**) per channel. Decay data analysis with a sum of exponentials was achieved by means of a Microsoft Excel spreadsheet specially designed for lifetime analysis that considers the convolution with the IRF [4]. The steady-state fluorescence anisotropies are defined by equation 2:

$$r = \frac{I_{\parallel} - I_{\perp}}{I_{\parallel} + 2I_{\perp}} \quad (2)$$

where $I_{\parallel}$ and $I_{\perp}$ are the fluorescence intensity observed with vertically polarized excitation light and vertically and horizontally polarized emission, respectively r values were determined using the *G*-factor method [5,6].

1. Brouwer, A.M. Standards for Photoluminescence Quantum Yield Measurements in Solution (IUPAC Technical Report). *Pure Appl. Chem.* **2011**, *83*, 12, 2213-2228.
2. Lakowicz, J.R. Principles of Fluorescence Spectroscopy, 3rd ed., Springer, New York, 2006.
3. Menezes, F.; Fedorov, A.; Baleizão, C.; Valeur, B. Berberan-Santos, M.N. Methods for the Analysis of Complex Fluorescence Decays: Sum of Becquerel Functions Versus Sum of Exponentials. *Methods Appl. Fluoresc.* **2013**, *1*, 015002.
4. Berberan-Santos, M.N. Unpublished. **2009**.
5. Chen, F.R., Bowman, L.R. Fluorescence polarization: measurement with ultraviolet-polarizing filters in a spectrophotofluorometer. *Science* **1965**, *147*, 729-732.
6. Valeur, B.; Berberan-Santos, M.N. *Molecular Fluorescence. Principles and Applications*, Wiley-VCH, 2nd ed., 2012, pp. 465-469.

*Crystallographic Data and Refinement Details of **2a**, **3a**, **3b** and **5a***

The asymmetric unit of the monoclinic crystals of **2a** (C2/c space group) (Figure **S3**) contains four crystallographically independent dihomooxacalix[4]arene molecules and 5.25 co-crystallised acetonitrile solvent molecules in six different sites, five at full occupancy (four located inside the calixarene cavities) and one with 0.25 occupancy. Two-position disorder was found for four tert-butyl groups, one for each independent molecule. One crystallographically independent molecule of **2a** also shows a two-position disorder of the complete phthalimide substituent, including the propyl linker. This disorder was refined with 0.67/0.33 partial occupancies. SIMU restraints were applied to the thermal factors of the lower occupancy phthalimide atoms.

The asymmetric unit of the monoclinic crystals of **3a** (C2/c space group) (Figure **S3**) contains three crystallographically independent dihomooxacalix[4]arene molecules, four co-crystallised acetonitrile solvent molecules (three located inside the calixarene cavities) and a half water molecule. Two-position disorder was found for a *tert*-butyl group on two different independent molecules. The other independent molecule of **3a** shows a two-position disorder (0.67/0.33 partial occupancies) of the complete phthalimide substituent, including the propyl linker. Two acetonitrile independent molecules show a two-position disorder of the C-N groups only, with the methyl carbon atoms superimposed. One molecule located inside the calixarene cavity was refined with 0.80/0.20 occupancy factors, with the other, external to the cavity, was refined with 0.67/0.33 occupancy factors.

The asymmetric unit of the monoclinic crystals of **3b** ($P2_1/c$ space group) (Figure **S3**) contains one crystallographically independent dihomooxacalix[4]arene molecules and three co-crystallised acetonitrile solvent molecules. The acetonitrile molecule hosted in the calixarene cavity shows a two-position disorder (0.80/0.20 partial occupancies) of the C-N group only, with the methyl carbon atoms superimposed.

The asymmetric unit of the triclinic crystals of **5a** (P-1 space group) (Figure **S3**) contains one crystallographically independent dihomooxacalix[4]arene molecules, 1.45 dichloromethane and 0.35 ethanol co-crystallised solvent molecules. Two different solvent sites are present in the crystal packing, one inside the cavity of the calixarene and one between the calixarene molecules. Both sites contain superimposed dichloromethane and ethanol solvent molecules with partially occupancy. The internal calixarene site was refined with 0.80 dichloromethane and 0.20 ethanol, while the external site was refined with 0.65 dichloromethane and 0.15 ethanol.

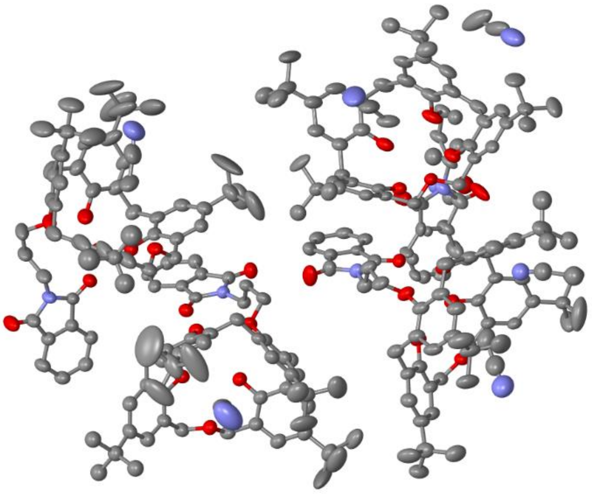

2a

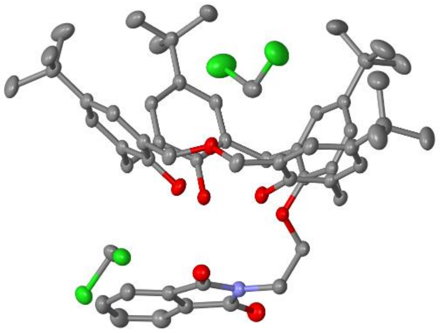

5a

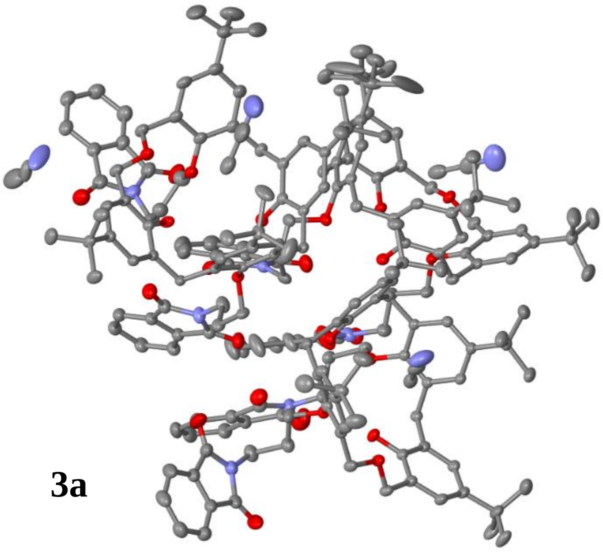

3a

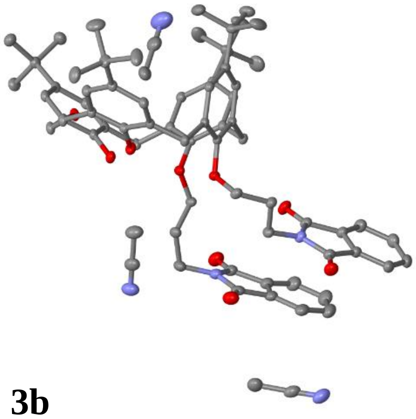

3b

Figure S3. Asymmetric units of **2a**, **3a**, **3b** and **5a**. The atomic species are represented in CPK colours. Ellipsoids are drawn at 50% probability, except for **2a**, in which the ellipsoids are represented at 30% probability. Hydrogen atoms and disordered fragments are omitted for clarity.

Table S4. Crystal data and structure refinement for compounds **2a**, **3a**, **3b** and **5a**

	**2a**	**3a**	**3b**	**5a**
Empirical formula	$C_{56}H_{67}NO_7$, 1.31(C_2H_3N)	$C_{67}H_{76}N_2O_9$, 1.33(C_2H_3N), 0.17(H_2O)	$C_{67}H_{76}N_2O_9$, 3(C_2H_3N)	$C_{55}H_{65}NO_7$, 1.45(CH_2Cl_2), 0.35(C_2H_6O)
Formula weight	919.99	1111.03	1176.45	991.34
Temperature (K)	100(2)	100(2)	100(2)	100(2)
Wavelength (Å)	0.7	0.7	0.7	0.7
Crystal system	Monoclinic	Monoclinic	Monoclinic	Triclinic
Space group	*C* 2/c	*C* 2/c	*P* 2_1/c	P -1
Unit cell dimensions (Å, °)	a =32.91(2)<br>b = 28.74(2)<br>c = 47.12(3)<br>α = 90<br>β = 102.96(1)<br>γ = 90	a =35.163(3)<br>b = 20.096(1)<br>c = 53.262(3)<br>α = 90<br>β = 95.151(8)<br>γ = 90	a = 24.749(2)<br>b = 14.546(1)<br>c = 19.676(1)<br>α = 90<br>β = 108.76(2)<br>γ = 90	a = 13.505(1)<br>b = 14.034(1)<br>c = 16.110(1)<br>α = 67.604(8)<br>β = 89.55(3)<br>γ = 75.302(7)
Volume (Å^3)	43430(50)	37485(4)	6707(1)	2717.1(4)
Z	32	24	4	2
ρ_{calcd} (g/cm^3)	1.126	1.181	1.165	1.212
μ (mm^{-1})	0.070	0.075	0.073	0.204
F(000)	15836	14280	2520	1056
Reflections collected	116851	360398	127997	76308
Independent reflections	31039 [R_{int} = 0.0963]	52023 [R_{int} = 0.0379]	18778 [R_{int} = 0.0273]	15865 [R_{int} = 0.0387]
Data/restr./para m.	31039/15/2591	52023/0/2480	18778/0/812	15865/6/662
GooF	1.045	1.033	1.049	1.035
Final *R* indices [I>2σ(I)]	R_1 = 0.0968, wR_2 = 0.2374	R_1 = 0.0534, wR_2 = 0.1409	R_1 = 0.0384, wR_2 = 0.1072	R_1 = 0.0627, wR_2 = 0.1702
R indices (all data)	R_1 = 0.1431, wR_2 = 0.2693	R_1 = 0.065, wR_2 = 0.151	R_1 = 0.0426, wR_2 = 0.111	R_1 = 0.0761, wR_2 = 0.1811
CCDC code	2057047	2057046	2057044	2057045

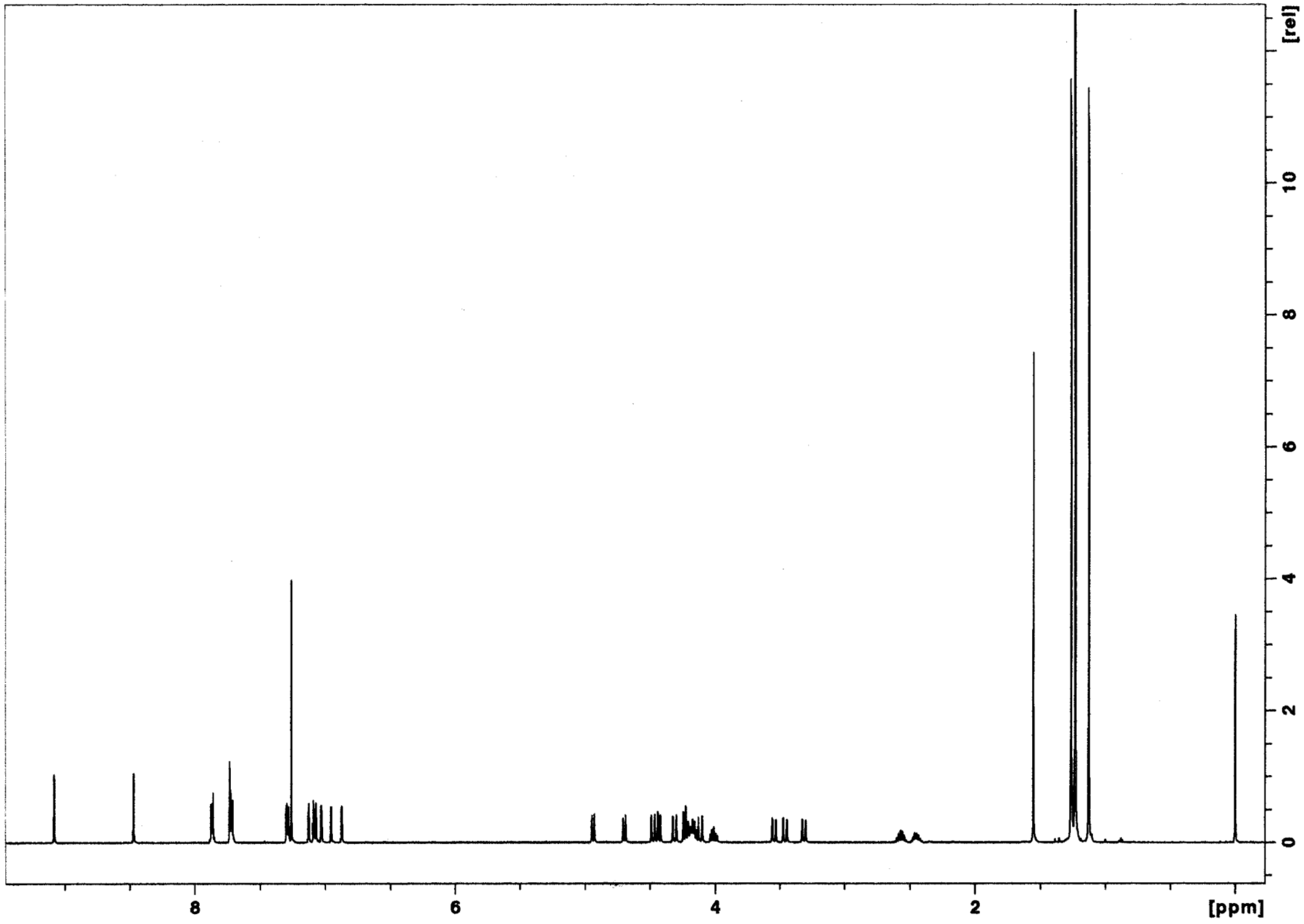

Figure S4. ^{1}H NMR spectrum (500 MHz, $CDCl_3$, rt) of monopropylphthalimide **2a**.

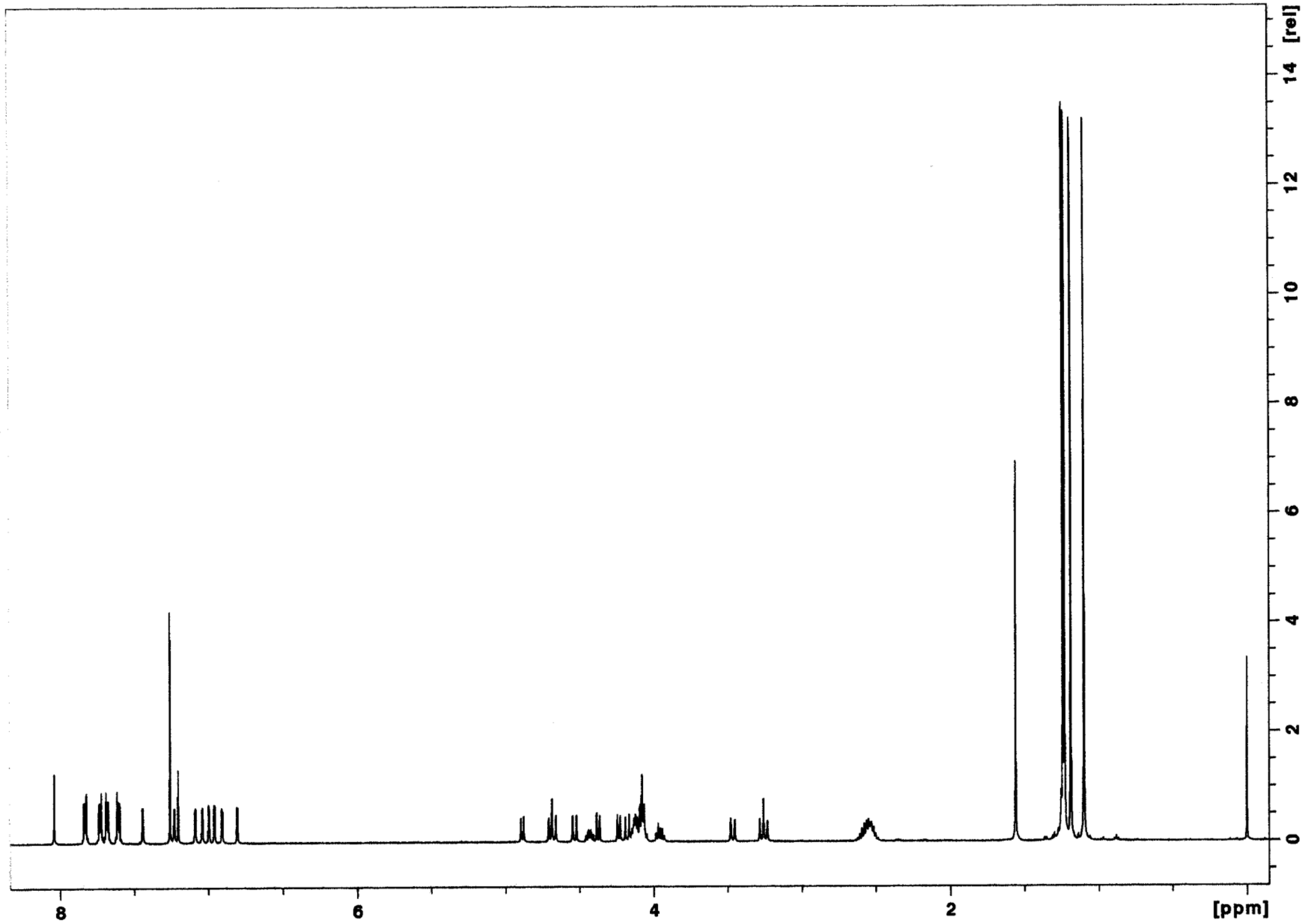

Figure S5. ^{1}H NMR spectrum (500 MHz, $CDCl_3$, rt) of 1,3-dipropylphthalimide **3a**.

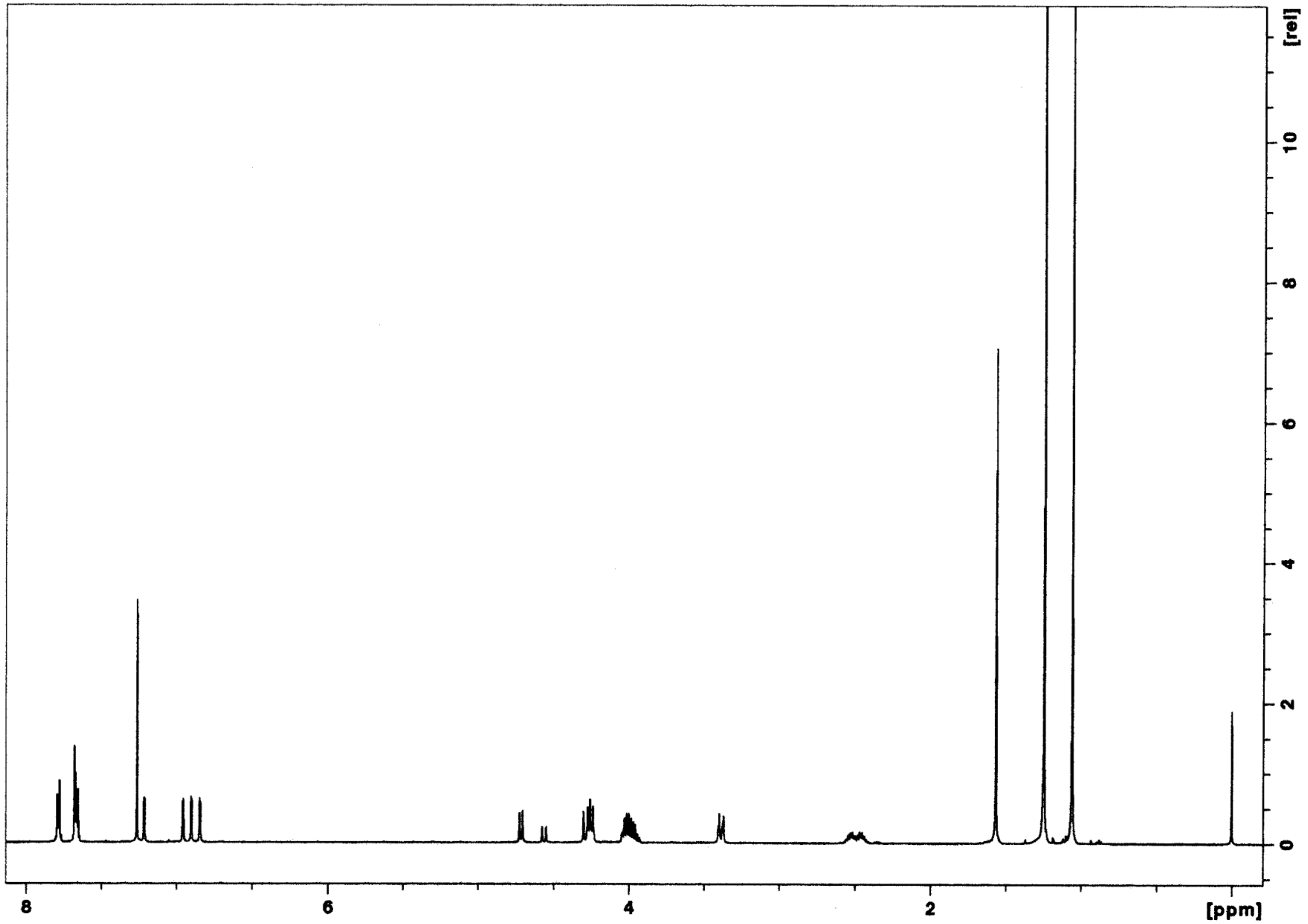

Figure S6. ^{1}H NMR spectrum (500 MHz, $CDCl_3$, rt) of 3,4-dipropylphthalimide **3b**.

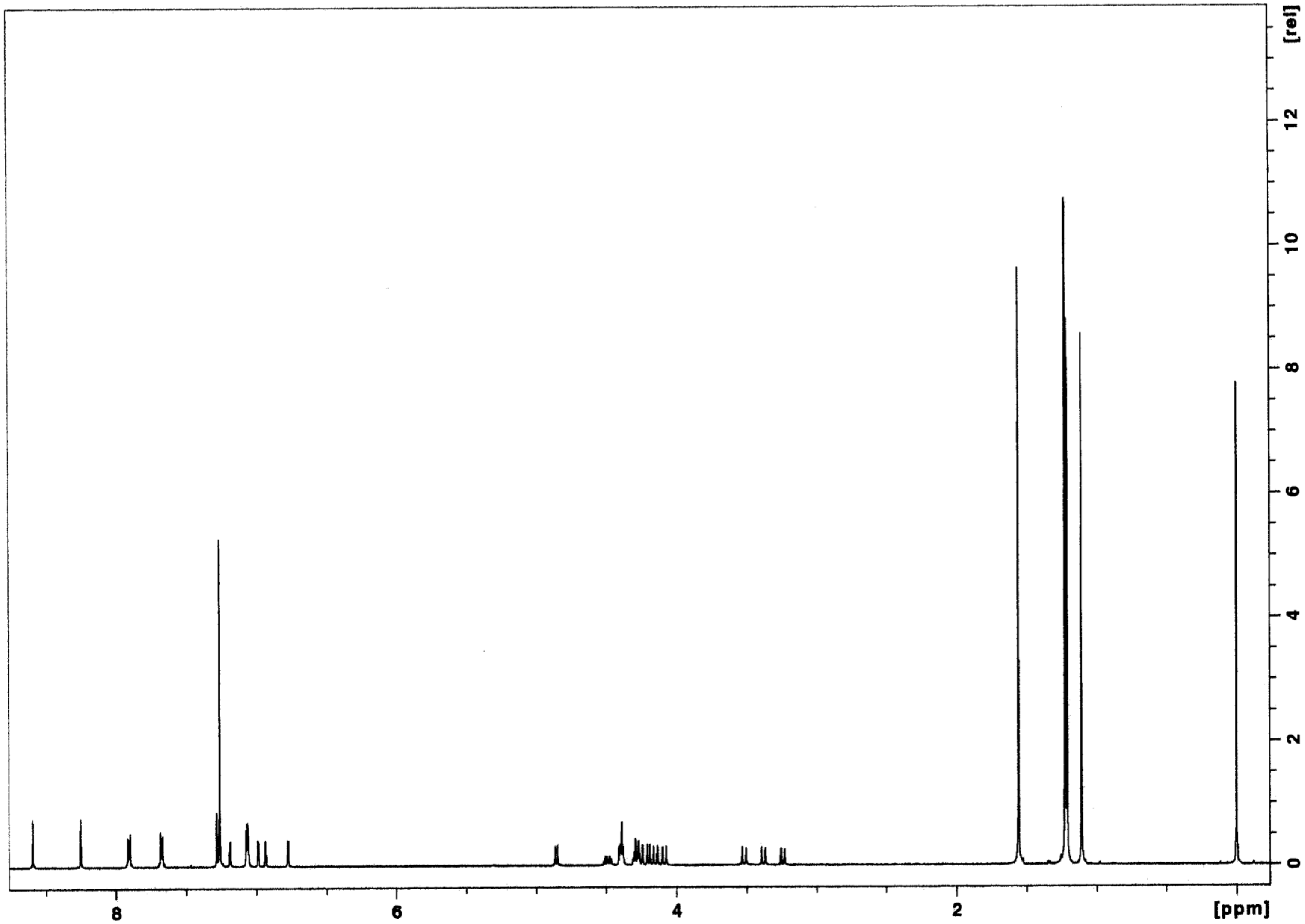

Figure S7. ^{1}H NMR spectrum (500 MHz, $CDCl_3$, rt) of monoethylphthalimide **5a**.

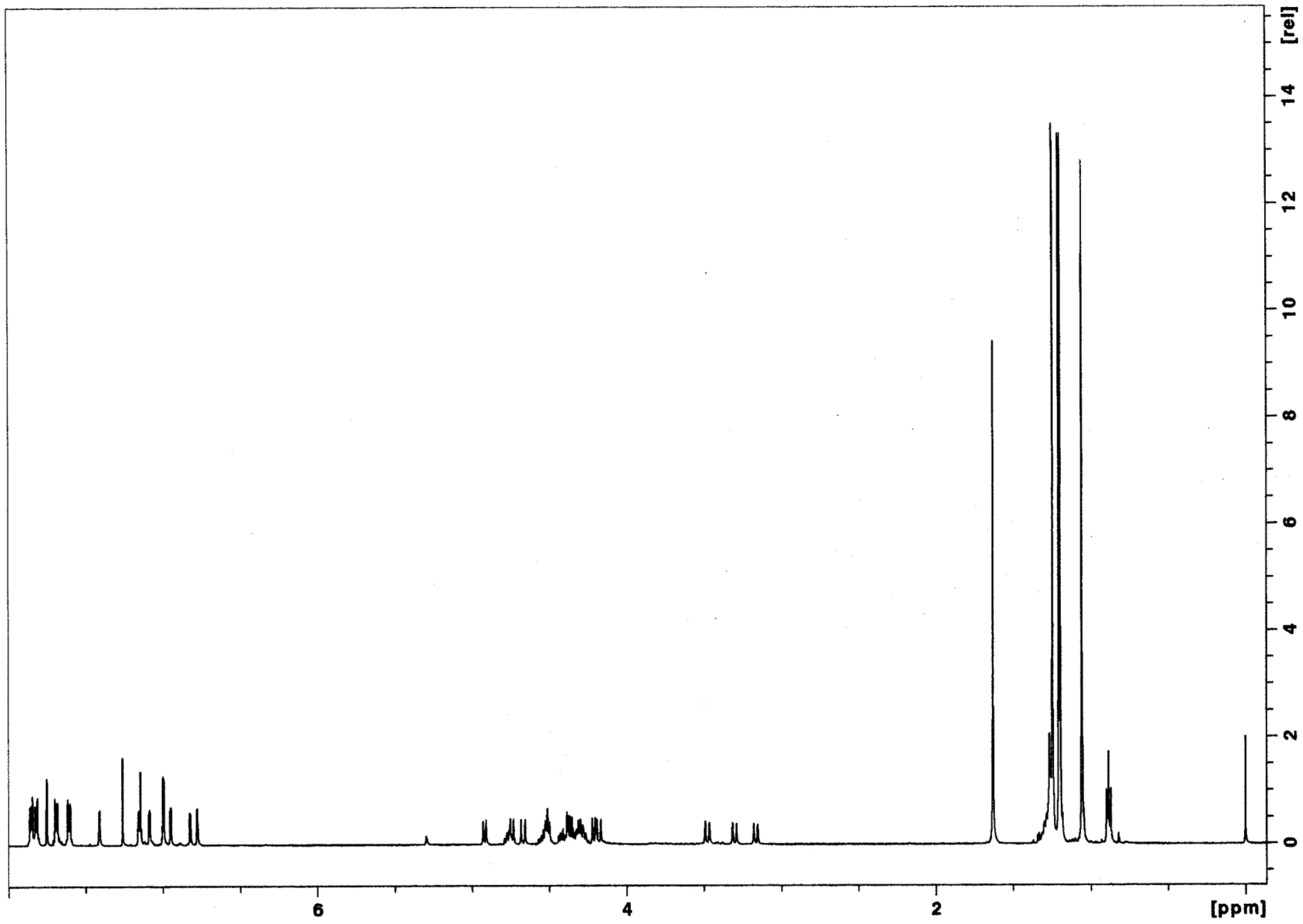

Figure S8. ^{1}H NMR spectrum (500 MHz, $CDCl_3$, rt) of 1,3-diethylphthalimide **6a**.

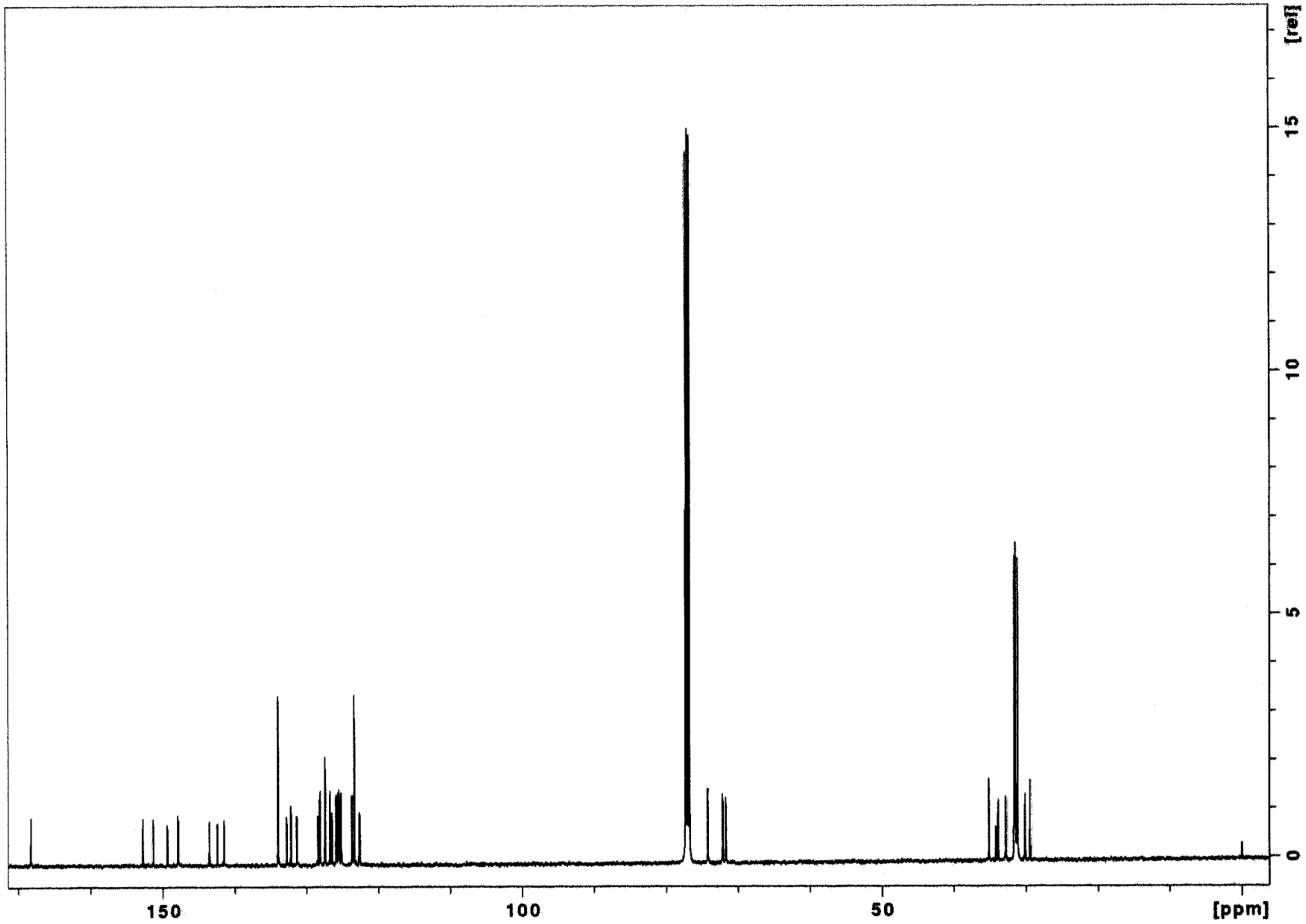

Figure S9. ^{13}C NMR spectrum (125.8 MHz, $CDCl_3$, rt) of monopropylphthalimide **2a**.

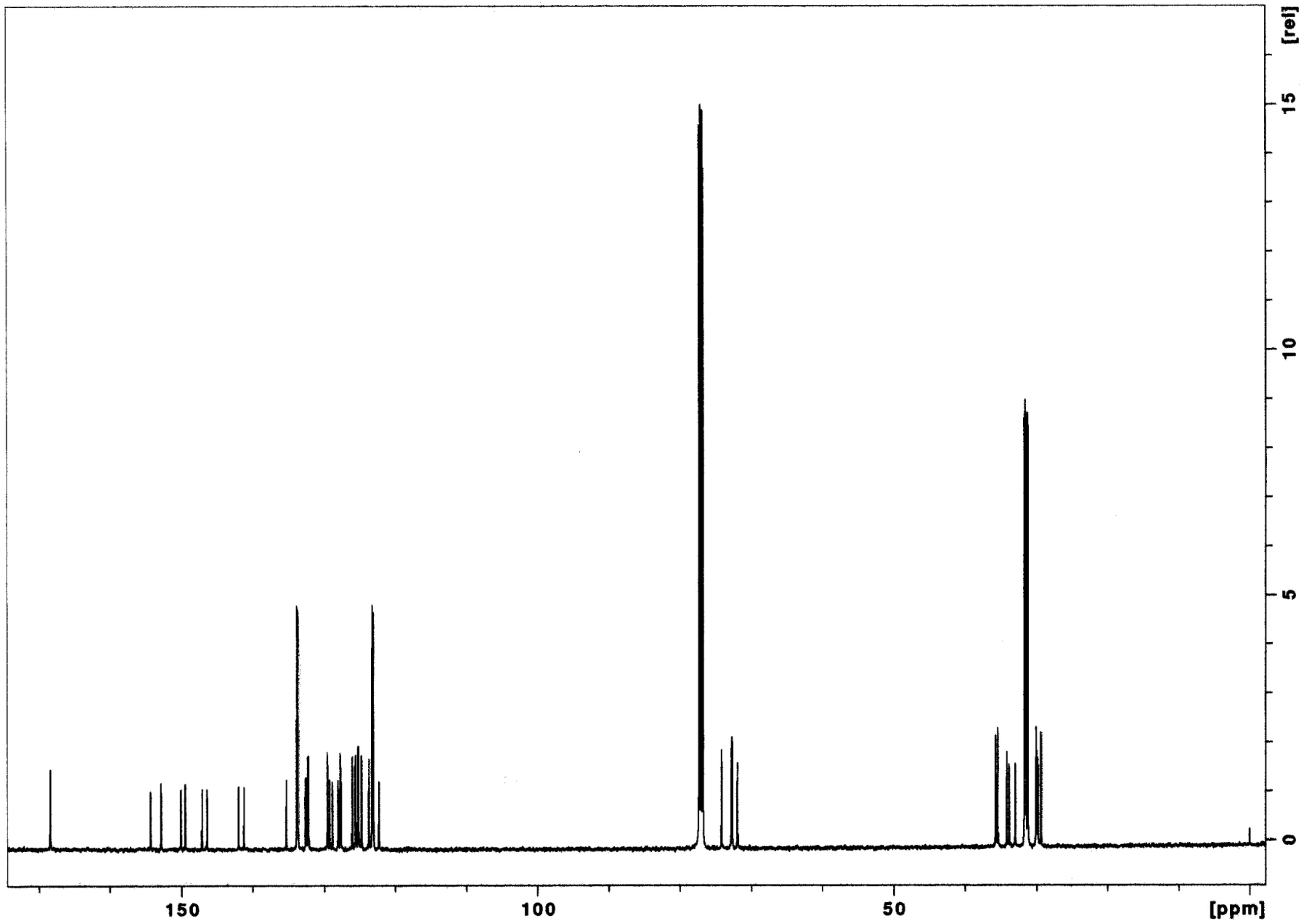

Figure S10. ^{13}C NMR spectrum (125.8 MHz, $CDCl_3$, rt) of 1,3-dipropylphthalimide **3a**.

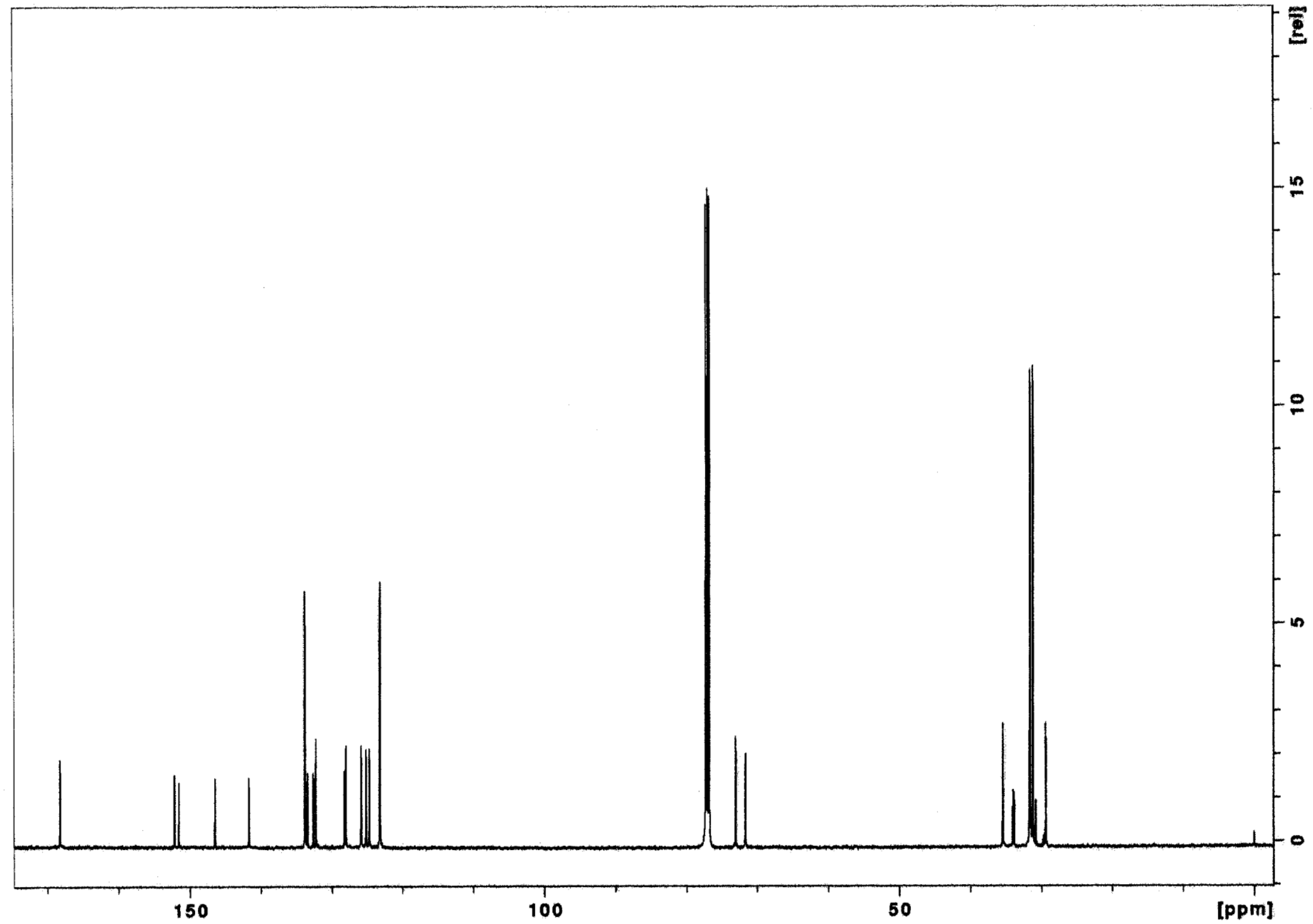

Figure S11. ^{13}C NMR spectrum (125.8 MHz, $CDCl_3$, rt) of 3,4-dipropylphthalimide **3b**.

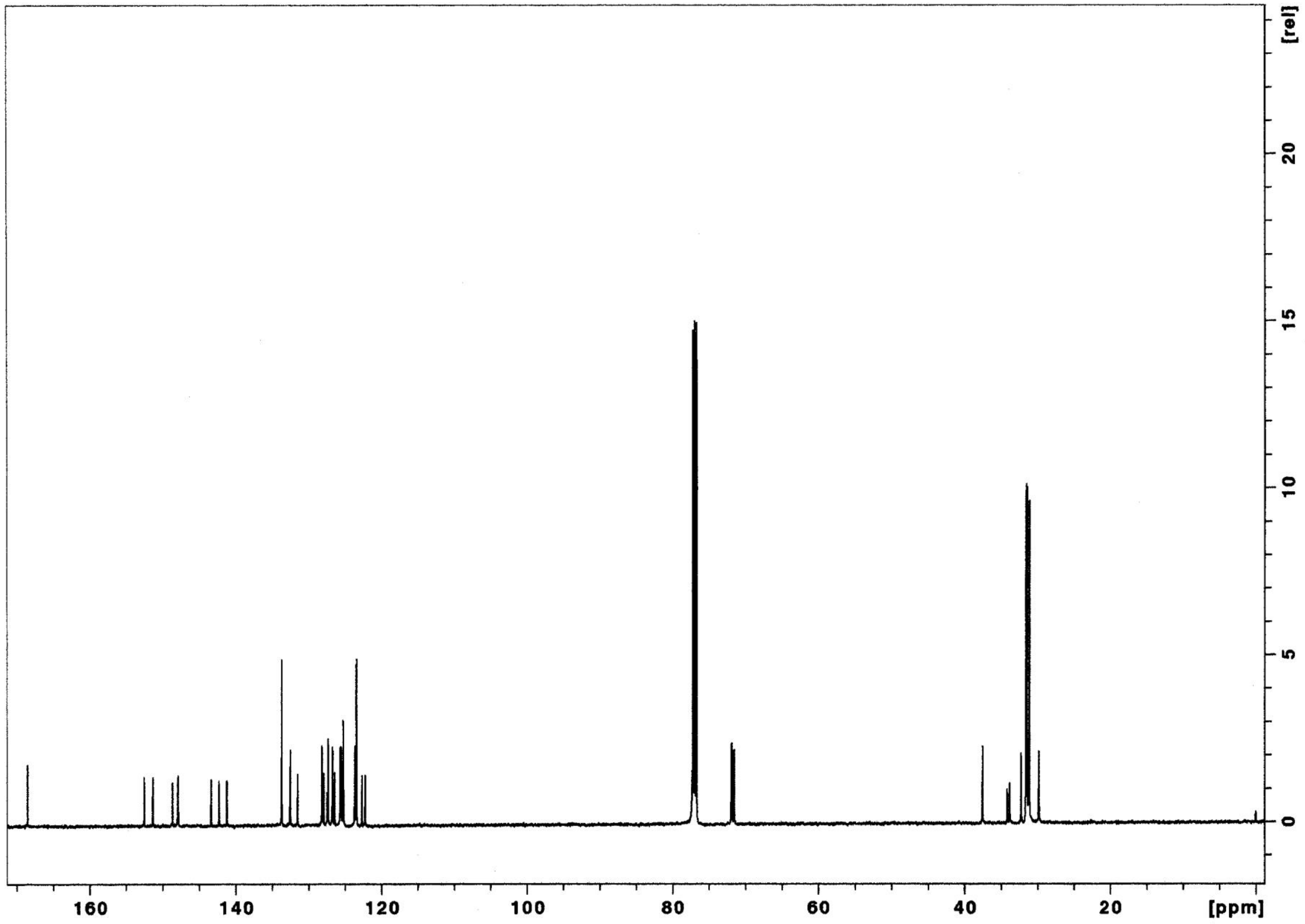

Figure S12. ^{13}C NMR spectrum (125.8 MHz, $CDCl_3$, rt) of monoethylphthalimide **5a**.

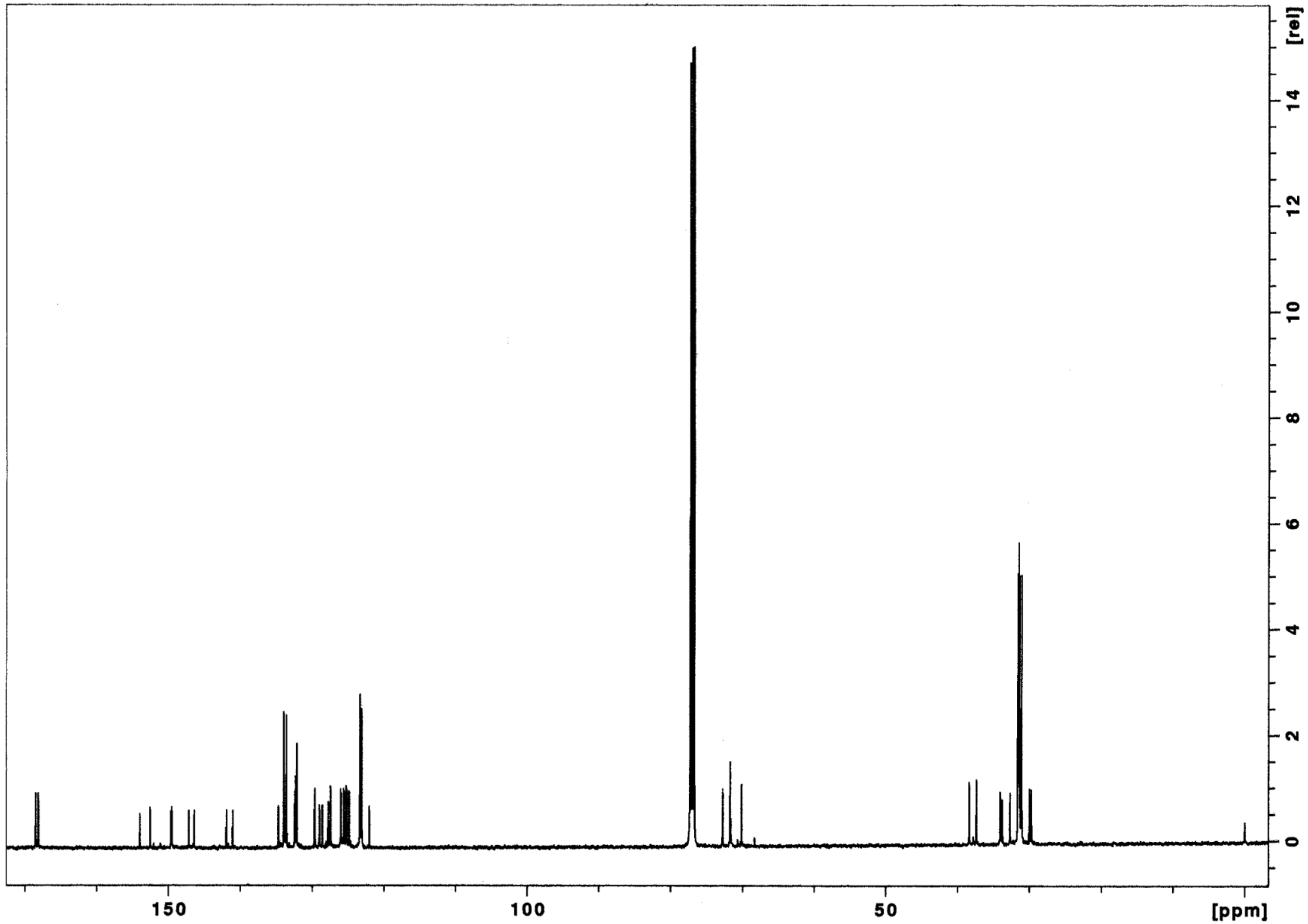

Figure S13. ^{13}C NMR spectrum (125.8 MHz, $CDCl_3$, rt) of 1,3-diethylphthalimide **6a**.

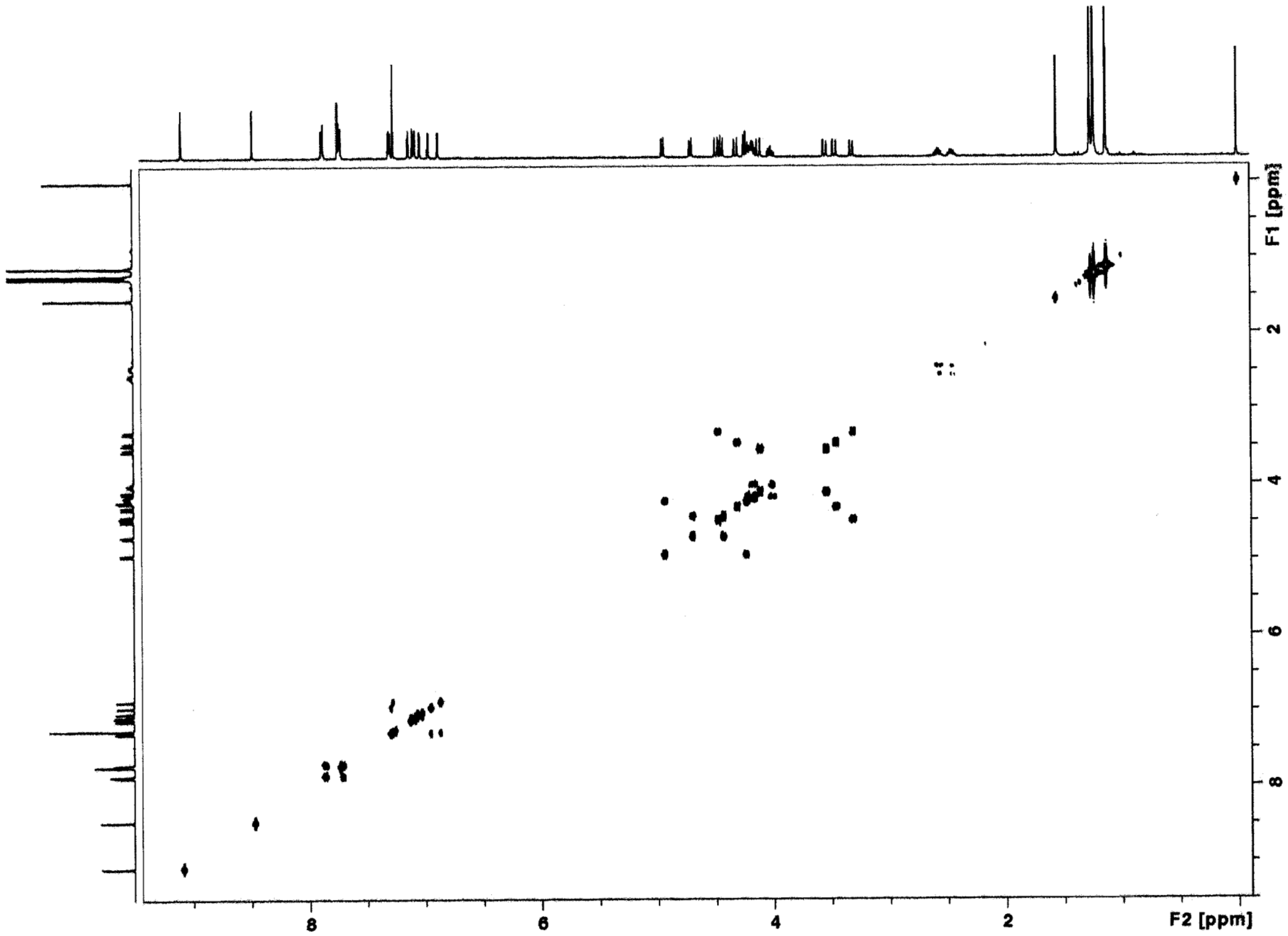

Figure S14. COSY spectrum (500 MHz, $CDCl_3$, rt) of monopropylphthalimide **2a**.

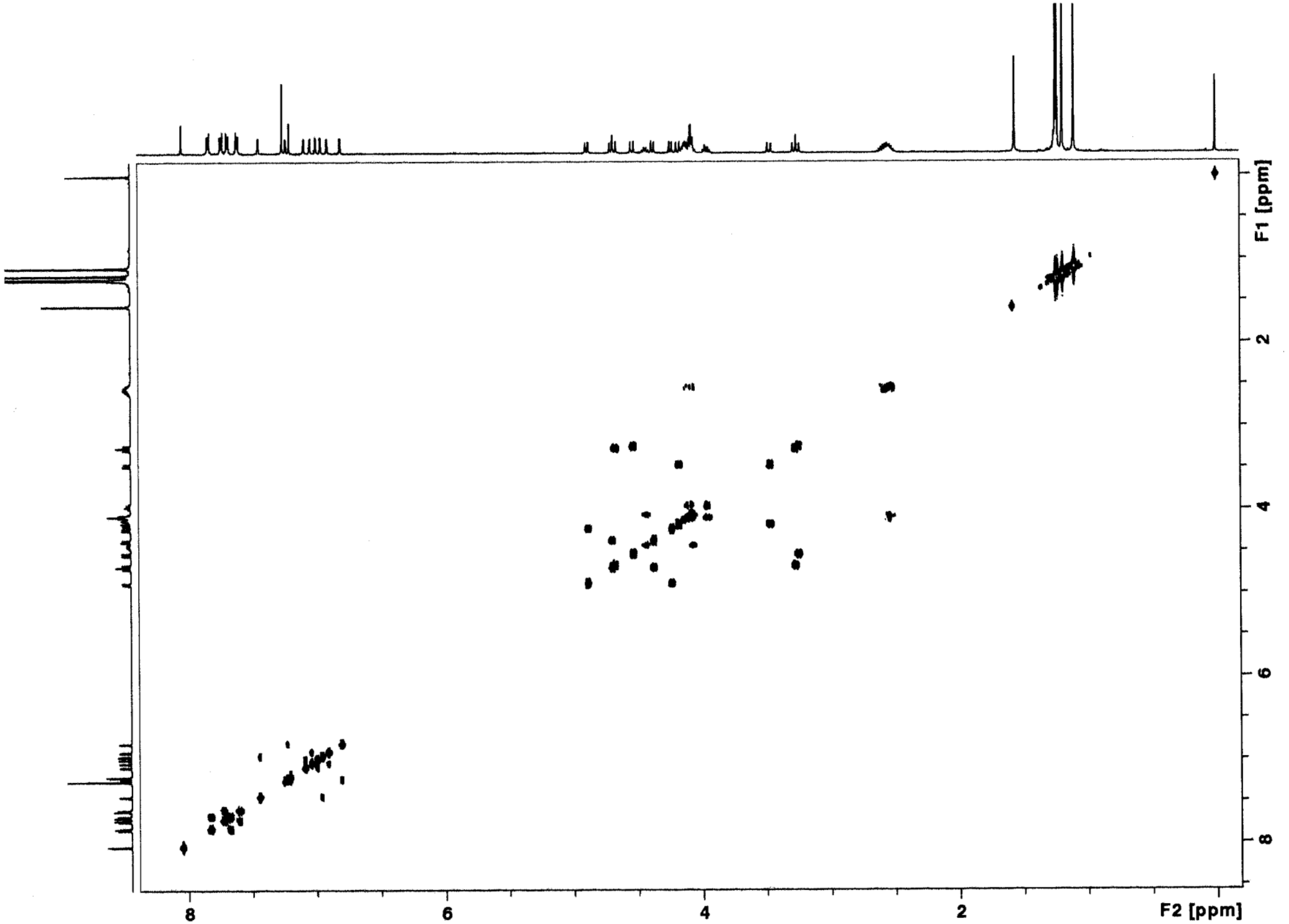

Figure S15. COSY spectrum (500 MHz, $CDCl_3$, rt) of 1,3-dipropylphthalimide **3a**.

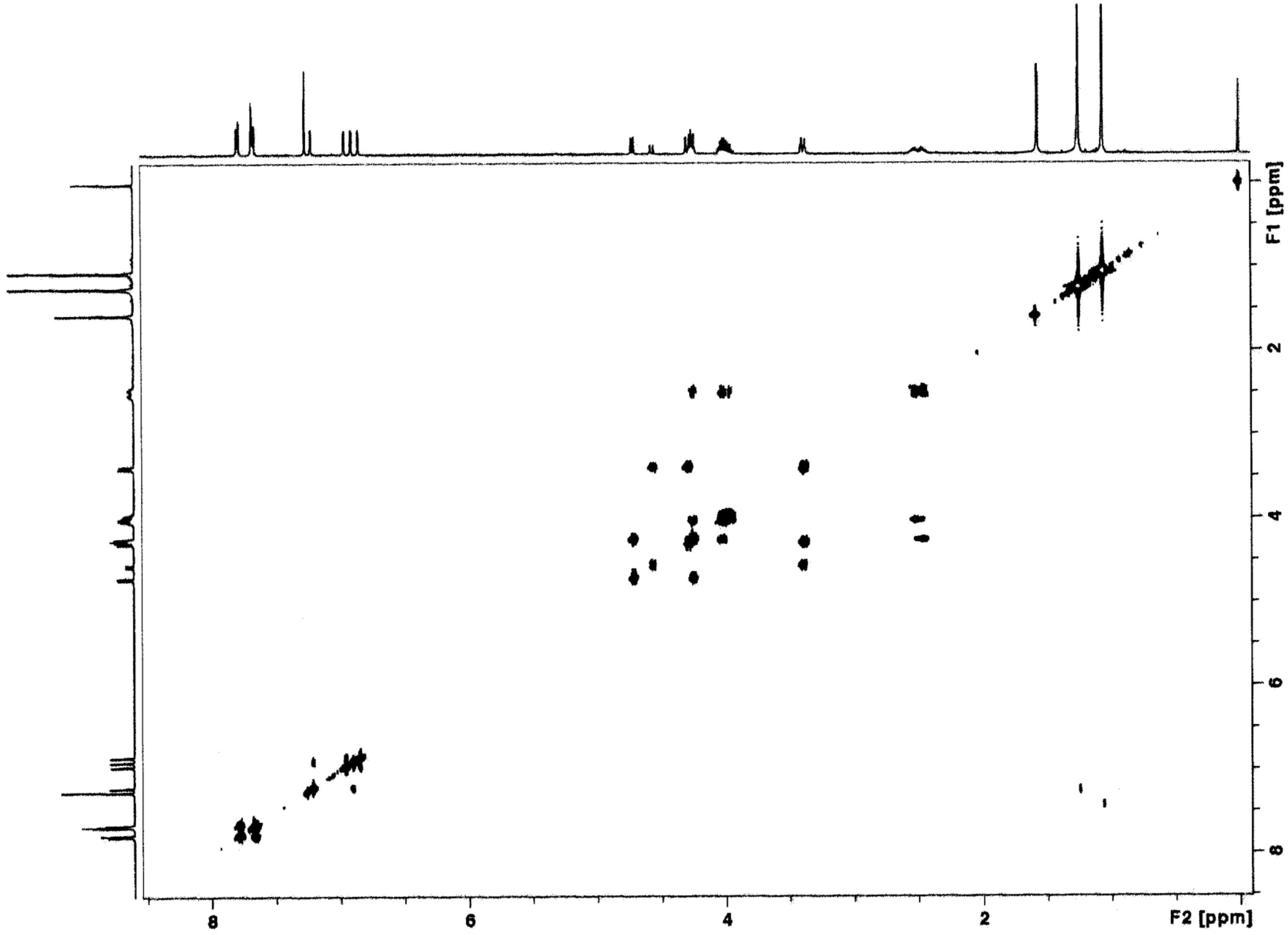

Figure S16. COSY spectrum (500 MHz, $CDCl_3$, rt) of 3,4-dipropylphthalimide **3b**.

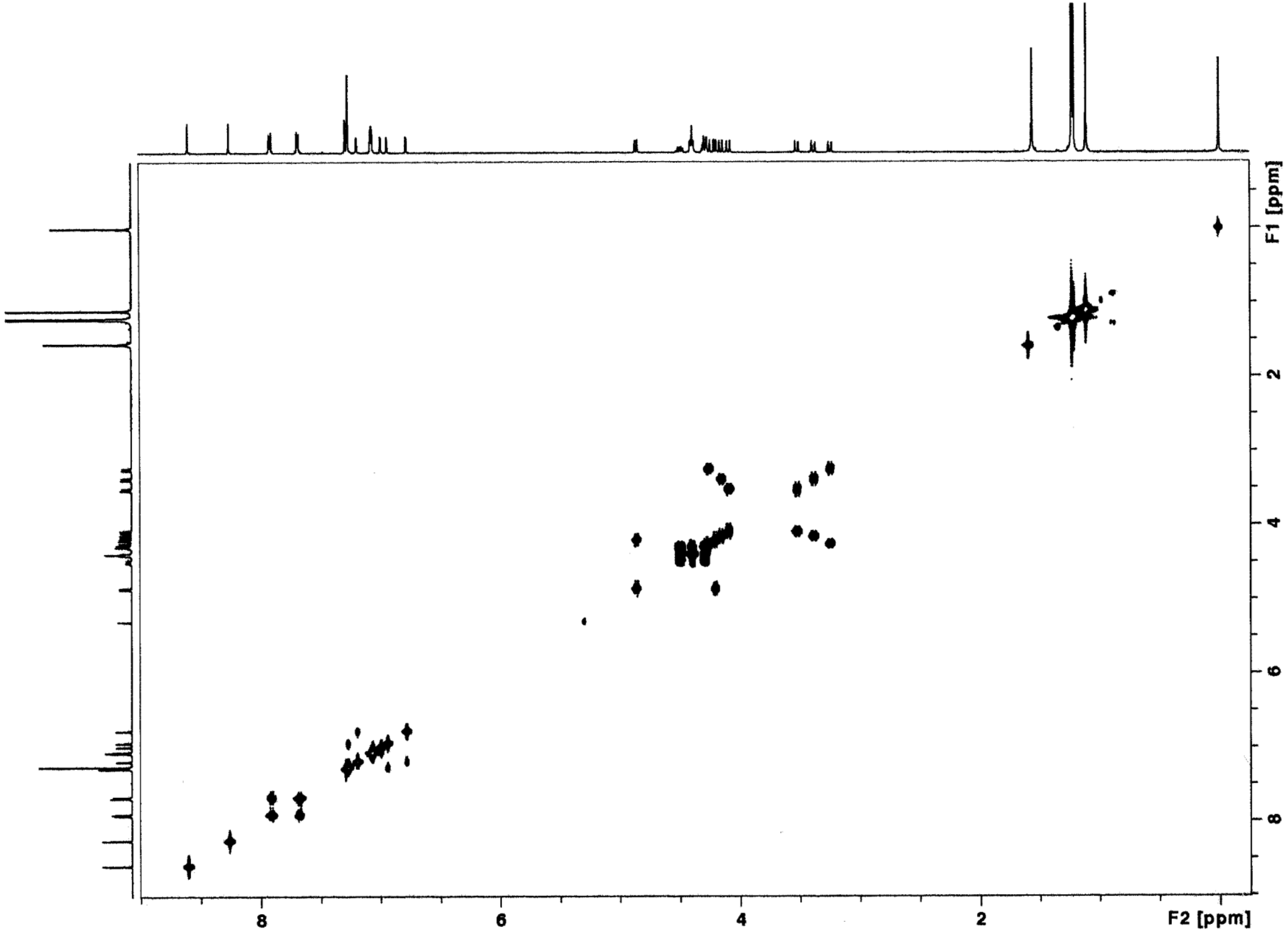

Figure S17. COSY spectrum (500 MHz, $CDCl_3$, rt) of monoethylphthalimide **5a**.

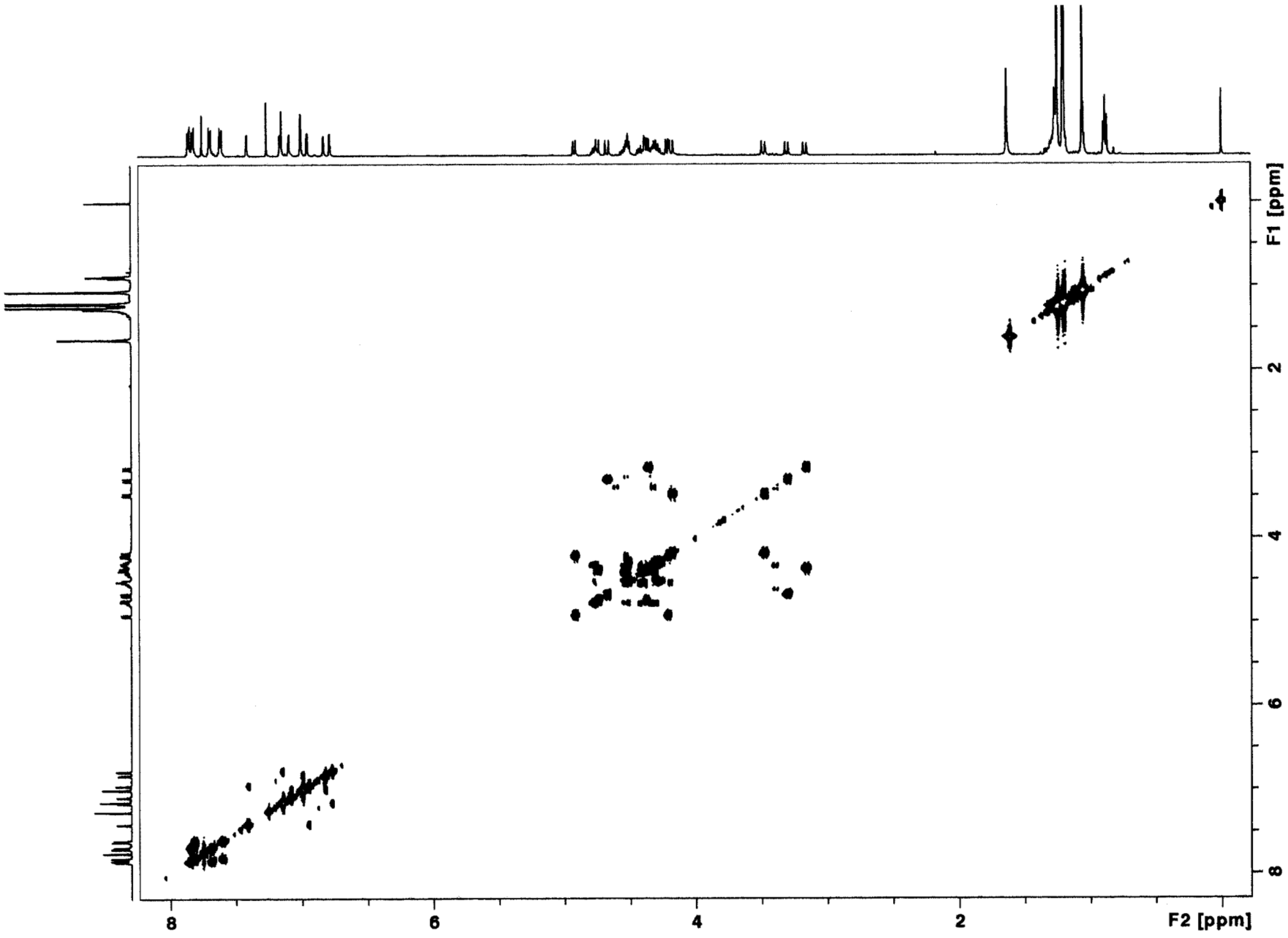

Figure S18. COSY spectrum (500 MHz, $CDCl_3$, rt) of 1,3-diethylphthalimide **6a**.

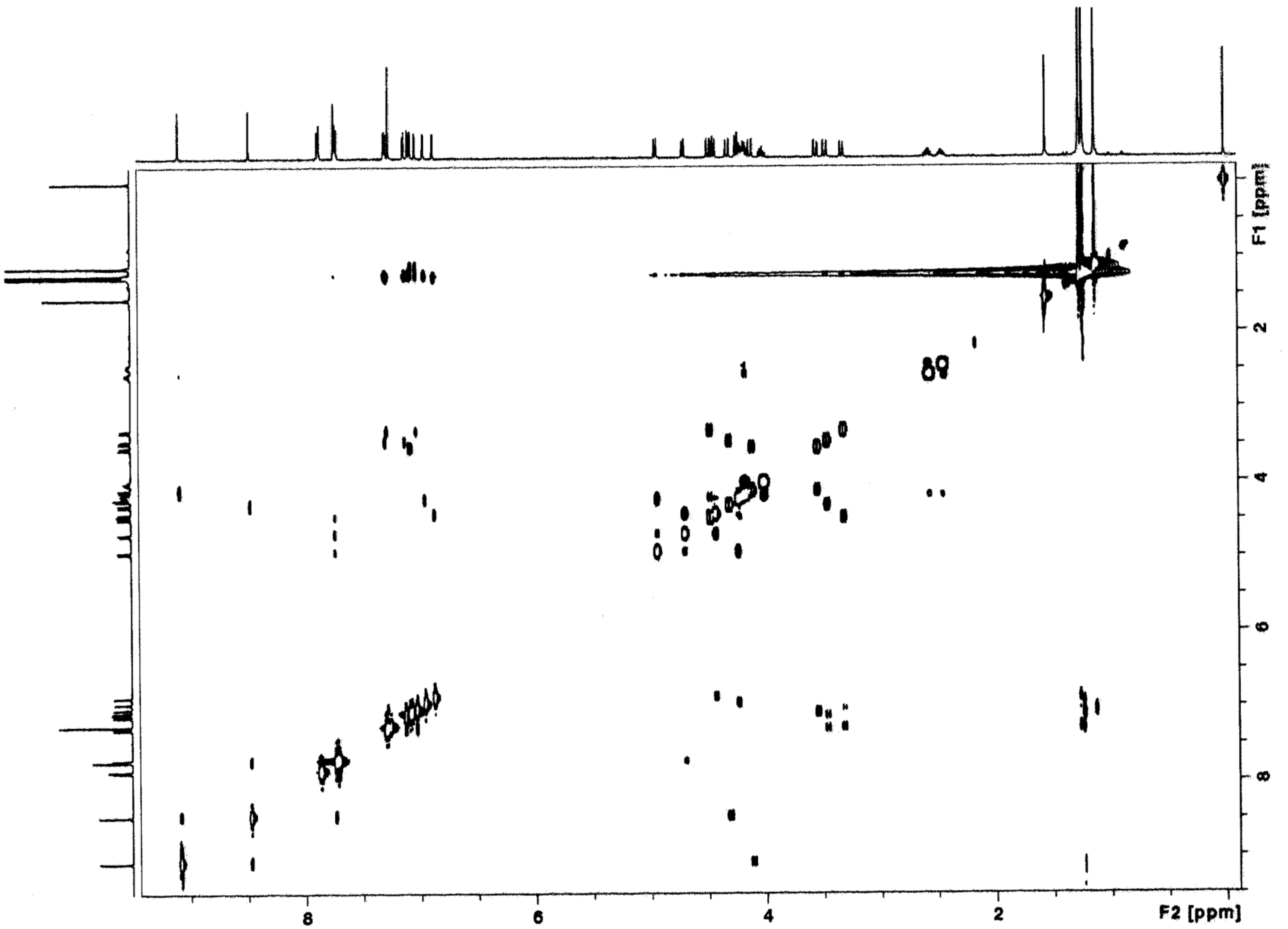

Figure S19. NOESY spectrum (500 MHz, $CDCl_3$, rt) of monopropylphthalimide **2a**.

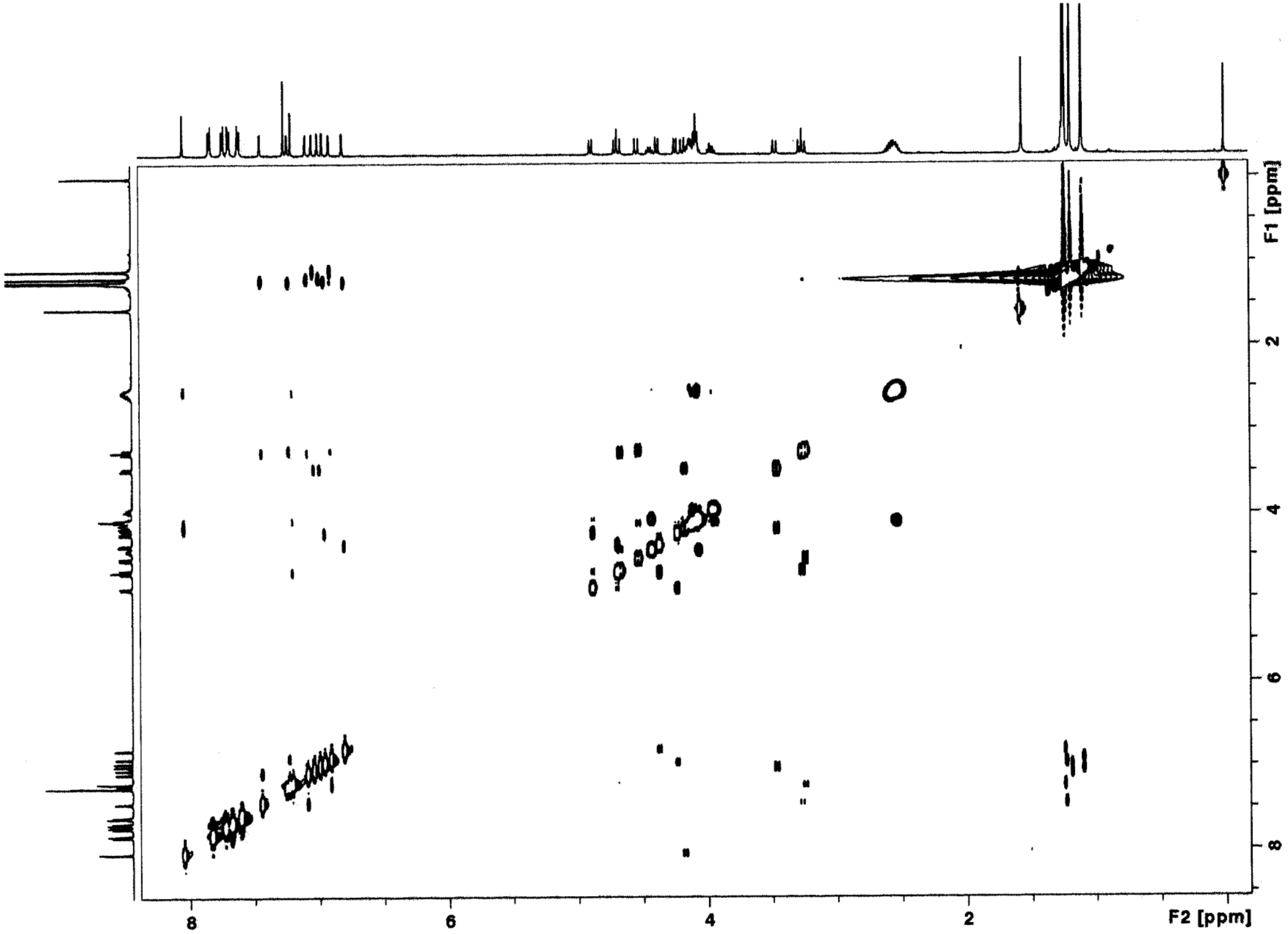

Figure S20. NOESY spectrum (500 MHz, $CDCl_3$, rt) of 1,3-dipropylphthalimide **3a**.

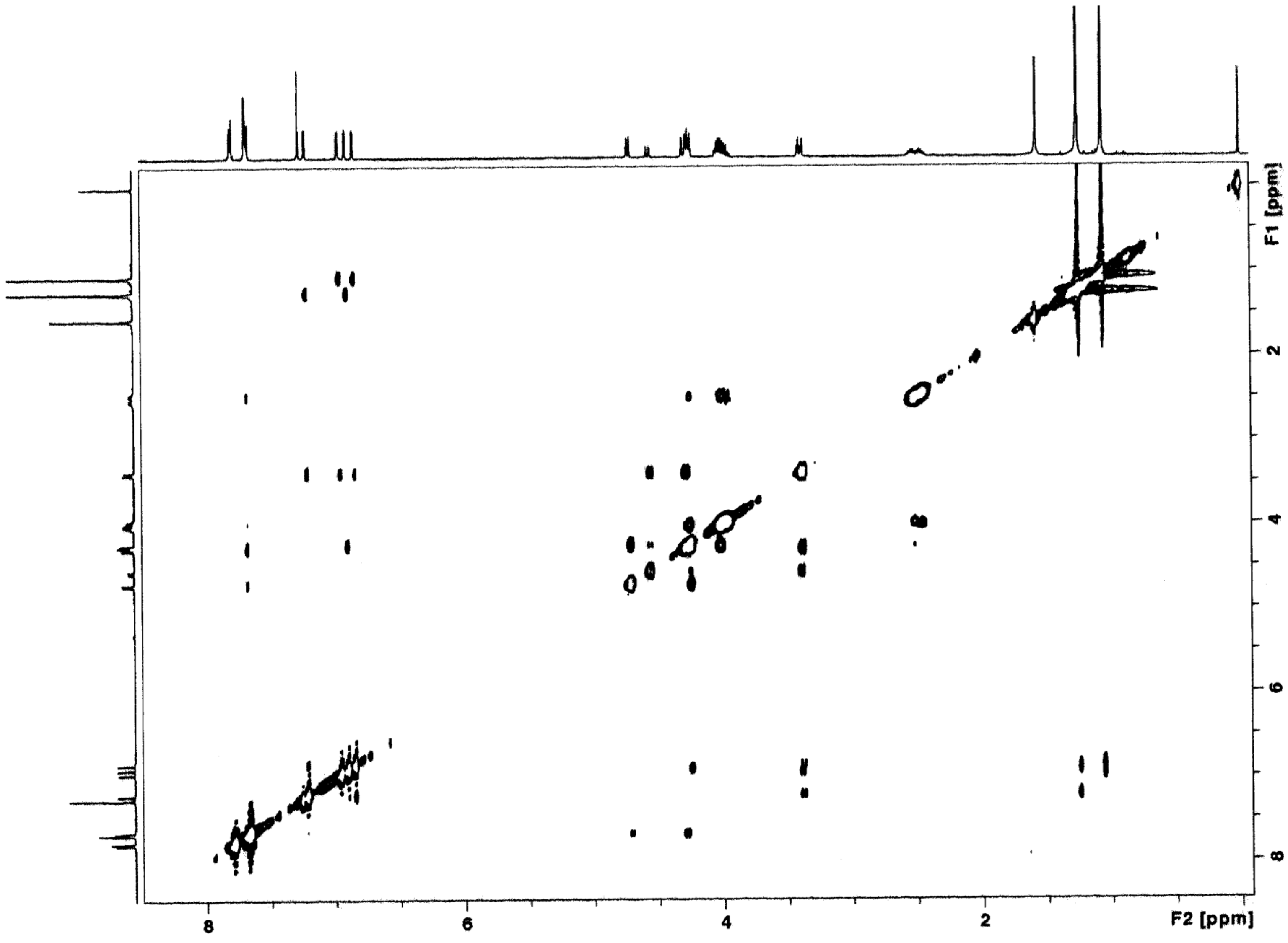

Figure S21. NOESY spectrum (500 MHz, $CDCl_3$, rt) of 3,4-dipropylphthalimide **3b**.

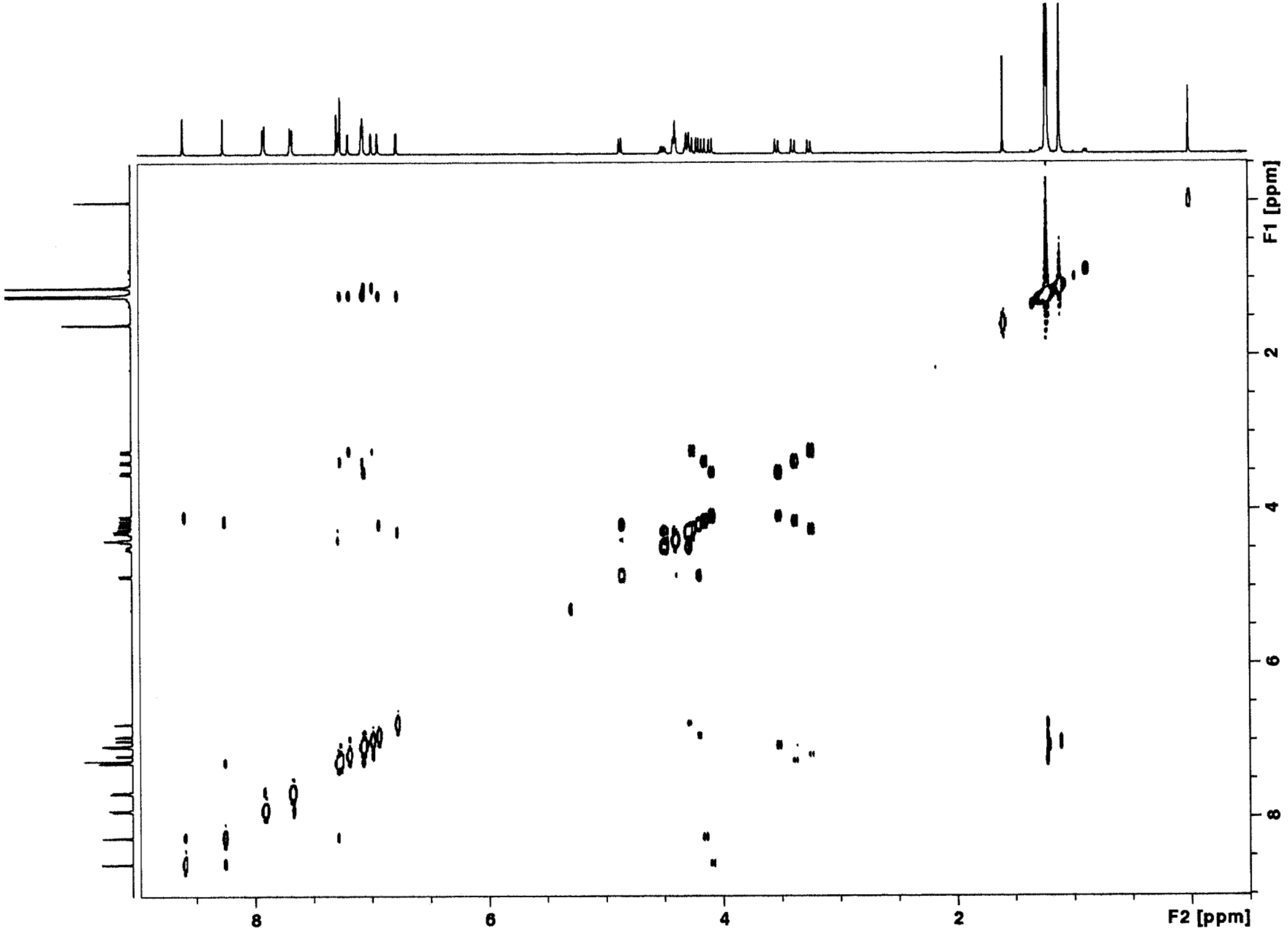

Figure S22. NOESY spectrum (500 MHz, $CDCl_3$, rt) of monoethylphthalimide **5a**.

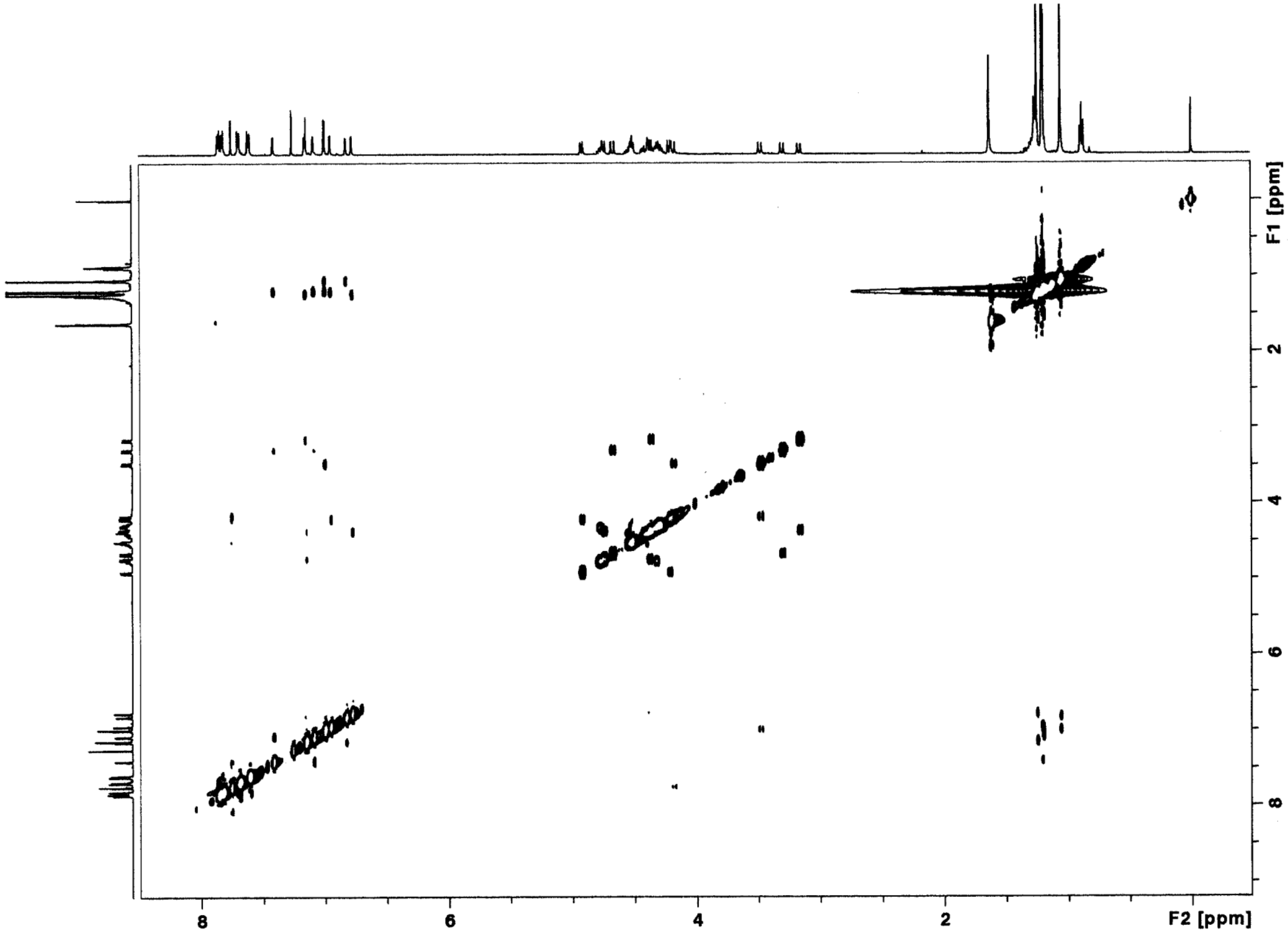

Figure S23. NOESY spectrum (500 MHz, $CDCl_3$, rt) of 1,3-diethylphthalimide **6a**.
